# Mechanisms of Taxane Resistance

**DOI:** 10.3390/cancers12113323

**Published:** 2020-11-10

**Authors:** Sara M. Maloney, Camden A. Spring, Lorena V. Morejon-Lasso, Jenifer R. Prosperi

**Affiliations:** 1 Harper Cancer Research Institute, South Bend, IN 46617, USA; smalone3@nd.edu; 2 Department of Biochemistry and Molecular Biology, Indiana University School of Medicine, South Bend, IN 46617, USA; 3 Department of Biological Sciences, University of Notre Dame, Notre Dame, IN 46556, USA; choover3@nd.edu (C.A.S.); lmorejon@nd.edu (L.V.M.-L.)

**Keywords:** breast cancer, prostate cancer, ovarian cancer, taxane resistance

## Abstract

**Simple Summary:**

Drug resistance is prevalent in many types of cancer and decreases patient survival. The taxanes are anti-mitotic chemotherapeutic agents, widely used since the 1990s to treat many types of cancer. Even with the popularity of the taxane family, many patients are, or will become, resistant to taxane treatment, meaning that other, perhaps less effective, treatment options are required. This review article seeks to provide information about the common cancers in which taxanes are used and resistance occurs, in order to find targetable mechanisms that can be used to overcome resistance.

**Abstract:**

The taxane family of chemotherapy drugs has been used to treat a variety of mostly epithelial-derived tumors and remain the first-line treatment for some cancers. Despite the improved survival time and reduction of tumor size observed in some patients, many have no response to the drugs or develop resistance over time. Taxane resistance is multi-faceted and involves multiple pathways in proliferation, apoptosis, metabolism, and the transport of foreign substances. In this review, we dive deeper into hypothesized resistance mechanisms from research during the last decade, with a focus on the cancer types that use taxanes as first-line treatment but frequently develop resistance to them. Furthermore, we will discuss current clinical inhibitors and those yet to be approved that target key pathways or proteins and aim to reverse resistance in combination with taxanes or individually. Lastly, we will highlight taxane response biomarkers, specific genes with monitored expression and correlated with response to taxanes, mentioning those currently being used and those that should be adopted. The future directions of taxanes involve more personalized approaches to treatment by tailoring drug–inhibitor combinations or alternatives depending on levels of resistance biomarkers. We hope that this review will identify gaps in knowledge surrounding taxane resistance that future research or clinical trials can overcome.

## 1. Introduction

### 1.1. Taxane Family of Chemotherapy

Taxanes (paclitaxel, docetaxel, and cabazitaxel) belong to the microtubule-stabilizing class of antimitotic cancer drugs. These drugs suppress microtubule (MT) dynamics by preferentially and reversibly binding to the β-subunit of the tubulin heterodimer [1]. MTs are involved in a variety of cellular processes, such as signaling, migration, and division, that are critical for cancer cell proliferation and metastasis [2]. By adding or removing tubulin subunits at the MT ends, MTs can alternate between growth and shortening phases through their characteristic “dynamic instability” behavior. Given that dynamic spindle MTs are vital for effective cell division, drugs that suppress MT dynamic instability can be useful to prevent cancer cell proliferation. Binding of taxanes stimulates microtubule polymerization and induces the formation of stable MT bundles. This action alters the natural dynamics of MTs, prevents proper spindle formation, blocks mitosis, and induces apoptosis [3].

#### 1.1.1. Paclitaxel

Paclitaxel (PTX) was the first drug of the taxane family to be discovered through a partnership between the National Cancer Institute (NCI) and the US Department of Agriculture (USDA). An extensive search for potential anticancer agents from natural products culminated in the discovery of PTX in the bark extract of the Pacific yew tree (*Taxus brevifolia*). The structure of PTX was later discovered to contain an eight-member taxane ring with a bulky ester side chain at C-13 deemed essential for antitumor activity [4]. After demonstrating efficacy in mouse models in 1978, the NCI confirmed the antitumor activity of PTX and selected the drug as a candidate for clinical development [5]. Taxol^®®^ achieved approval by the US Food and Drug Administration (FDA) in 1992, becoming the first taxane drug available for cancer treatment [6]. Since then, a variety of alternative PTX formulations have been developed as well as second and third generations of the drug. However, it was later found that taxanes are poorly water-soluble drugs and do not sufficiently dissolve when administered in their crystalline form [7,8]. Moreover, the chemical solvent Cremaphor EL required for PTX administration induced adverse side effects and cytotoxicity on its own [9]. This led to the development of nanoparticle albumin-bound PTX (nab-PTX), which added albumin protein nanoparticles to PTX and enabled it to enter the bloodstream and cells, where it separates from the nano-albumin and induces G2-M arrest [10]. The improved drug delivery system increases intratumoral concentrations while shielding normal, healthy cells from drug toxicities. Clinical trials have shown success with nab-PTX in metastatic breast and pancreatic cancer [11,12], though more research should be done on potential benefits in other cancer types.

#### 1.1.2. Docetaxel

Before the semi-synthetic version was created, conservation concerns over the large numbers of Pacific yews required for PTX clinical doses led to a large-scale search for alternative sources of the drug [13,14]. Ultimately, a compound extracted from the faster-growing European yew tree, *Taxus baccata*, was developed into the semi-synthetic drug docetaxel (DTX). DTX differs structurally from PTX in the 3’-position of the lateral chain and the 10-position on the taxane ring. Although taxanes share the same or overlapping binding sites, DTX and PTX also differ in the length of intracellular drug retention and the strength of their suppressive effect on MT dynamics [2]. DTX generates tubulin polymers that differ structurally from those produced by PTX and has been demonstrated to have greater efficiency in tubulin assembly by binding to β-tubulin with greater affinity [15,16]. Evidence also suggests that DTX is retained longer within cancer cells compared to PTX [15]. Shown to be approximately twice as potent as PTX, the drug has since been widely used after its FDA approval in 1996 [17].

#### 1.1.3. Cabazitaxel

Due to drug resistance frequently limiting the usefulness of PTX and DTX, the novel taxane drug cabazitaxel (CBZ) was developed as a semi-synthetic dimethyloxy derivative of DTX [12]. CBZ differs from DTX in that its methyl groups at the 7- and 10-positions of the taxane ring drastically reduce its affinity for the ATP-binding cassette (ABC) drug efflux pump, MDR1. CBZ also exhibits more prolonged intracellular retention and more significant suppression of MT dynamics compared to DTX. As a result, CBZ produces a more enduring G2-M arrest and induces a more substantial apoptotic effect [18]. Studies have shown that CBZ retains activity in DTX-resistant tumors, perhaps because of the proposed mechanisms mentioned above [19]. CBZ was approved by the FDA in 2010 as a second-line treatment for metastatic castration-resistant prostate cancer (mCRPC) patients who have already demonstrated resistance to a DTX-containing treatment regimen [14]. Further research is needed to explore the potential benefit of CBZ-based treatment as a first-line therapy.

### 1.2. The Problem of Taxane Resistance

**Figure 1:**
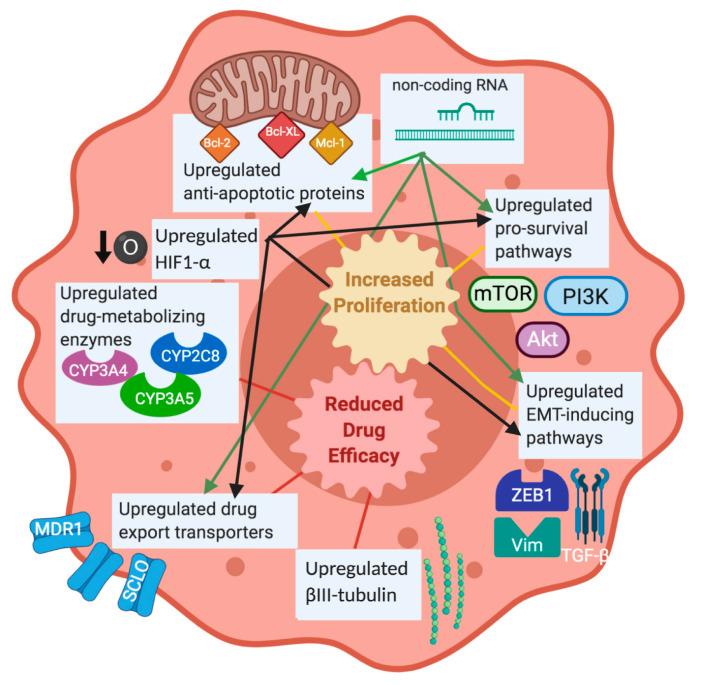
Overview of taxane resistance mechanisms. This diagram illustrates some of the major proteins and pathways that are known to contribute to taxane resistance in cancer. Upregulated pro-survival pathways, induction of EMT through upregulated vimentin, ZEB1, and TGF-β, and upregulated anti-apoptotic proteins have been linked with increased proliferation after taxane treatment. Upregulated tubulin isotypes, drug export transporters, and drug-metabolizing enzymes have been associated with reduced taxane efficacy. Hypoxia and non-coding RNAs have been noted to activate some of the established resistance mechanisms, so they will be considered to be contributing factors to taxane resistance as well. The review will include these mechanisms and others, which are frequently observed in different cancer types. Figure was made in BioRender.

Despite the excitement that taxanes brought to the chemotherapy drug field, specifically PTX, resistance is often observed in patients. Taxane resistance is multifactorial, involving a variety of mechanisms and genes that either work alone or in conjunction with other factors to inhibit taxane function. Research has identified some of the mechanisms of taxane resistance; however, the full scope of taxane resistance has yet to be elucidated. Some of the common methods of taxane resistance that will be discussed herein are included in Figure 1 and are listed in Table 1. The methods of taxane resistance that are unique to specific cancers are listed in Table 2. Non-coding RNAs have been associated with activating or inhibiting key cellular pathways involved in survival and apoptosis, making them a driving force in the development of resistance to chemotherapy, including taxanes. Lists of non-coding RNAs are summarized by cancer type in Table 3, highlighting the shared RNAs between different cancer types and those unique to each cancer type.

#### 1.2.1. Taxane-Metabolizing Enzymes

Cytochrome P450 (CYP) enzymes are responsible for the degradation of over 60% of marketed drugs including PTX and DTX [20]. DTX is primarily metabolized by CYP3A5 and PTX by CYPC8, while both drugs can be metabolized by CYP3A4. While CYP enzymes are present in both healthy and malignant tissue, upregulation of these enzymes has been observed in cancerous tissue and may be a major factor in the pathogenesis of several cancers including breast, prostate, lung, ovarian, and endometrial cancer. A correlation between CYP enzymes and chemoresistance could imply that CYP and drug metabolism limit efficacy or contribute to the development of resistance to taxane therapy by limiting intracellular drug concentrations [7,20]. Drug metabolism and increased metabolites have low levels of antitumor and cytotoxic activity. However, there are some challenges to inhibiting CYP enzymes to prevent taxane metabolism. Without active CYP enzymes, other drugs may not be metabolized to their inactive form and present unexpected or potentially dangerous drug–drug interactions [21]. In addition, the cytotoxicity of xenobiotics could increase or the activation of prodrugs could not occur without detoxification and bioactivation mediated by CYP enzymes [22].

#### 1.2.2. ATP-Dependent Pumps

Alterations in drug transport, especially in drug efflux, are a common mechanism of chemoresistance in cancer. One of the most well-known causes of multidrug resistance (MDR) in tumor cells is the overexpression of P-glycoprotein (P-gp), an ATP-binding cassette (ABC) drug efflux pump encoded by the MDR1 gene [23,24]. Overexpressed P-gp lowers intracellular drug concentration by accelerating drug efflux, making it a common mechanism of drug resistance [25]. PTX and DTX are both substrates of P-gp [26,27]. Though P-gp remains a consistent challenge to overcoming resistance, use of chemical inhibitors has resulted in challenges and limited clinical success. Because of its binding domain, P-gp is a very difficult target to design specific inhibitors for and could require higher doses with increased side effects [28]. In addition, other membrane drug pumps could compensate for P-gp interference, making the inhibitor irrelevant [29]. Still, P-gp upregulation is a major mechanism in taxane resistance and more research is required to better understand drug export.

#### 1.2.3. Tubulin Subunit Expression

Taxanes work exclusively with tubulin in MTs to prevent polymerization and depolymerization, but if different forms of tubulins with different binding affinities to taxanes are overexpressed, the anti-mitotic response could be reduced. There are multiple isotypes of tubulin, βI-, βII-, βIII-, βIVa-, and βV. Notably, the overexpression of βIII-tubulin has been linked to taxane resistance in multiple cancers [30,31,32]. As opposed to MTs composed of a variety of β-tubulins, MTs purified to contain only βIII-tubulin show greater dynamicity, as evidenced by a greater overall rate of exchange of tubulin dimers with MT ends [33]. A higher ratio of bound PTX is thus required in these MTs for stabilization [34]. βIII-tubulin also binds significantly less to taxanes than the other forms of tubulin due to a single amino acid difference [35]. Therefore, upregulation of βIII tubulin may disrupt MT dynamics and has been associated with DTX, PTX, and CBZ resistance in multiple cancer types [36,37,38,39,40,41,42].

While cancer is deadly, observed resistance post-treatment has profound effects: aggressive disease that is refractory to treatment is responsible for 90% of the deaths among patients for advanced ovarian cancer [43]. Likewise, of those who are diagnosed with early breast cancer, an estimated 1 out of 3 will eventually develop recurrent or metastatic disease, with a median 5-year survival of less than 25% [44]. Though the 5-year survival rate of men with prostate cancer has improved, all patients eventually develop resistance to treatment [45]. In this review, we will describe the major taxanes (PTX, DTX, and CBZ) used clinically and focus on the methods of taxane resistance that occur in breast, prostate, and ovarian cancers.

#### 1.2.4. Hypoxia Response Pathway

The cellular response to low oxygen levels (hypoxia) induces pathways responsible for cell survival, proliferation, autophagy, and stemness and can contribute to taxane resistance [46]. Upregulation of the key transcription factor hypoxia-induced factor 1-α (HIF1-α) is observed in many cancer types [47], but its increased expression alone has only been correlated with poor prognosis and not development of taxane resistance [48]. HIF1-α altered proliferation proteins and pathways, including cell cycle-driving cyclins, TGF-β and JNK [49,50], help to reduce the efficacy of taxane-induced G2-M arrest and apoptosis. To survive in adverse oxygen conditions, the tumor cell also decreases natural pro-apoptotic mechanisms, marked by a HIF1-α interaction with members of the BCL-2 family [51]. This would therefore allow for decreased taxane-mediated apoptosis. Upregulation of HIF1-α also induces expression of proteins related to epithelial-mesenchymal transition (EMT) and cell stemness, which decrease taxane efficacy and increase resistance to taxane-induced apoptosis [50]. Lastly, the increased oxygen usage with no replenishment observed in poorly vascularized solid tumors leads to decreased cellular metabolism and forces tumors to find alternative fuels and reactions via autophagy [52]. Hypoxia is not in itself a driving mechanism of taxane resistance but, because the signal response activates multiple already established resistance mechanisms, it should be mentioned in this review. There are some pre-clinical inhibitors targeting HIF1-α synthesis, activation, and function reviewed in [53,54,55], yet their lack of specificity has led to disappointing clinical findings. More research is required to fully understand the complexities of the hypoxia response and develop hypoxia-activated prodrugs to increase specificity and efficacy.

## 2. Taxane Resistance in Breast Cancer

Breast cancer (BC) is the most commonly diagnosed malignancy and the leading cause of cancer death in women worldwide [56]. While chemotherapy can improve survival rates and quality of life, as few as half of BC patients may benefit from chemotherapy due to resistance [57,58]. The issue of resistance is especially problematic for patients with triple-negative breast cancer (TNBC), a subtype of breast cancer characterized by the lack of HER2 amplification, and estrogen and progesterone receptors. This results in an inability to use targeted therapies, such as Tamoxifen or Herceptin to target ER+ BC or HER2+ BC, respectively. As a result, chemotherapeutics such as taxanes are the standard of care for TNBC treatment, but drug resistance remains a major obstacle affecting many patients [59,60]. Therefore, further exploration of the mechanisms underlying drug resistance will allow for the identification of biomarkers and development of novel therapies to overcome taxane resistance in BC.

### 2.1. Drug Transport and Efflux

Substantial evidence exists for the role of P-gp in mediating taxane resistance in vitro; however, demonstrating its role in vivo has proven more difficult [61,62,63]. Nevertheless, ongoing research continues to explore the possibility of P-gp inhibition as a sufficient means for overcoming taxane resistance in vivo. An herbal extract, SH003, was shown in MCF-7 cells to inhibit STAT3, which then blocks transcriptional activation of MDR1 [64]. Multiple studies have demonstrated that MAPK proteins, such as ERK and p38, are involved in regulation of MDR1 expression and therefore taxane resistance in breast cancer. In addition to the above-mentioned regulation by STAT3, MDR1 expression is also under transcriptional control of EGR1, which is regulated by ERK1/2 [65]. A taxane-like compound, NPB304, sensitizes PTX-resistant cells in vitro and in vivo through inhibition of ERK and p38. In addition, NPB304 increases PTX accumulation through alterations of MDR1 [66]. Protein kinase D2 (PKD2), which is linked to MAPK, also regulates PTX response via MDR1 expression in MDA-MB-231 cells [67].

While the majority of the literature has focused on MDR1, loss of ABCC10, another ATP-dependent drug efflux pump, resulted in increased sensitivity to DTX and PTX in vivo and in vitro [68]. This suggests that other ATP-dependent pumps may have compensatory functions in taxane efflux and should be considered in the search for targeted therapies.

### 2.2. Drug Metabolism

In addition to drug transport, alterations in drug metabolism can also lead to taxane resistance. Cytochrome p450 (CYP) is a family of metabolizing enzymes which includes CYP3A4, the enzyme mainly responsible for PTX and DTX metabolism [69,70]. BC patients with lower CYP3A4 mRNA expression exhibited a significantly higher response rate (71%) to DTX than those with low CYP3A4 mRNA expression (response rate, 11%) [71]. Another study looking at CYP3A4 expression in BC by immunohistochemistry found similar results [72]. These data suggest that CYP enzymes can serve as a good predictive marker for therapeutic response to taxanes.

### 2.3. Alteration of Microtubule Regulatory Proteins and Tubulin Isotypes

Altered expression of MT-associated proteins (MAPs) and other proteins that affect MT behavior may provide insight into alterations of cell cycle progression and other potential mechanisms of drug resistance.

#### 2.3.1. Microtubule (MT) and MT Dynamics

β-tubulin isotype expression plays a pivotal role in many taxane-resistant breast cancers. Although most evidence points to βIII-tubulin as the most influential isotype, other isotypes have also been shown to regulate taxane resistance. The miR-100 family was identified by miRNA superarray as a link to alterations in β-tubulin isotype mRNA and/or protein expression as a result of drug treatment. The miR-100 tumor suppressor is significantly decreased in BC primary tumors and cell lines and further decreased upon PTX treatment in MCF-7 cells [73,74]. mRNA of *TUBB2A* and *TUBB3,* the genes encoding β-tubulin IIA and III, increased 2–3-fold upon PTX treatment but was decreased after miR-100 transfection [74].

MAPs and other proteins involved in MT dynamics are important markers of MT-targeting drug resistance in breast cancer [75]. MAP4 stabilizes MTs by raising the rescue frequency and plays a role in mitotic MT dynamics, thereby causing PTX resistance [76,77]. Previous studies have shown that MAP4 is inversely regulated by p53, which resulted in enhanced MT polymerization and taxane sensitivity in the C127 mammary cell line [78]. Given this information, p53-mediated downregulation of MAP4 may be a potential mechanism of taxane resistance. Tau is a MAP that enables tubulin polymerization and promotes MT stabilization [79]. Preincubation of tubulin with tau protein reduces PTX binding and PTX-induced MT polymerization [80]. Lowered tau expression could serve as a biomarker to determine which patients will benefit from PTX treatment, as it makes MTs more vulnerable to PTX and BC cells more sensitive to the drug. In addition, inhibiting tau function may be a useful therapeutic method to improve PTX response [80].

The septin family of GTPases spatially guides the direction of MT plus-end movement through suppression of MT catastrophe [81]. They also play an important scaffolding role in membrane compartmentalization and protection against protein degradation, emerging as potential mediators of chemoresistance and vital organizers of MAPs and cancer-associated signaling pathways [82]. Overexpression of septins, particularly septin 9, in MDA-MB-231 cells increased PTX resistance [83]. This resistance was enhanced by long-chain tubulin polyglutamylation and linked to altered MT dynamics and early relocalization of septin filaments from actin fibers to MTs.

Tubulin Binding Cofactor C (TBCC) is a protein responsible for proper folding of α and β-tubulin subunits into the MT [84]. *TBCC* overexpression resulted in increased soluble, non-polymerizable tubulins and decreased soluble, polymerizable dimers and a slight decrease in the tubulin content of MTs. In human BC cells overexpressing TBCC, MT dynamicity was lowered, and cell cycle distribution was altered such that a higher proportion of these cells was in the G2-M phase and a lower proportion in the S phase. These TBCC overexpressing variants showed increased PTX sensitivity, potentially caused by lower levels of MT dynamicity and the increase in target cells (cells in G2-M) for anti-proliferative drugs [85,86].

#### 2.3.2. MT Regulators in Mitosis and Cell Cycle Progression

Regulation of MT functions at the mitotic spindle are critical for functionality of the taxanes, and disruptions of these functions provide avenues for taxane resistance. NIMA-related Kinase 2 (NEK2) is a regulator of centrosome separation, which is a prerequisite for mitotic spindle assembly [87,88]. Both NEK2 and LIN9, the transcriptional regulator of NEK2, are elevated with taxane resistance in TNBC cells [87,89]. Inhibition of either NEK2 or LIN9 expression restored drug sensitivity by inducing mitotic errors and apoptosis. Combination treatments of NEK2 or LIN9 inhibitors and taxanes are proposed to improve BC patient outcomes [87,89].

Both stathmin and G Protein Signaling Modulator 2 (GPSM2/LGN) regulate the mitotic spindle and the G2-M phase of the cell cycle [75,90,91,92]. Consequently, alterations in stathmin or GPSM2/LGN could lead to impaired mitotic spindle function and taxane resistance [75,93]. The knockdown of *GPSM2* increases the resistance of BC cells to PTX both in vitro and in vivo [93]. Phosphorylation prevents stathmin from binding to tubulin, effectively controlling stathmin’s ability to impact MT stabilization [94]. Expression of mutant, non-phosphorylated stathmin led to G2-M cell cycle arrest, while overexpressing stathmin in BC cells led to reduced MT polymerization and reduced PTX binding [95,96]. This finding reveals a possible mechanism of stathmin-induced PTX resistance in BC.

G Protein-Coupled Receptor Kinase 5 (GRK5) regulates MT nucleation at the centrosomes and normal cell cycle progression [97]. GRK5 forms a signaling complex with, and activates, histone deacetylase 6 (HDAC6) by phosphorylating it at Ser-21. Transient knockdown of *GRK5* in MDA-MB-231 cells increased sensitivity to PTX, potentially through blunted HDAC6 activity and higher α-tubulin acetylation [98].

### 2.4. Non-Coding RNAs

Multiple proteins or pathways that lead to increased survival or reduced apoptosis can be regulated by short non-coding RNA, known as microRNAs (miRNAs or miRs), or long non-coding RNAs (lnc-RNAs).

#### 2.4.1. MicroRNAs (miRs)

Overexpression of miRs can lead to taxane resistance. miR-18a is abnormally overexpressed in TNBC and is further overexpressed in taxane-resistant TNBC [99,100]. In the TNBC cell lines, MDA-MB-231 and MDA-MB-468, PTX resistance was accompanied by miR-18a overexpression and decreased expression of Dicer, a component of miRNA processing machinery [99]. *miR-663* or *miR-3646* overexpression in breast cancer cells confers taxane resistance, which is rescued by miR-663 downregulation or transfection of miR-3646 inhibitors ([101,102] and reviewed in [103]). miR-125b is upregulated in PTX resistant BC cells compared to sensitive cell lines. Overexpression of *miR-125b* inhibits PTX-induced cytotoxicity in all cell lines studied. Mechanistically, miR-125b inhibits PTX-induced apoptosis by suppressing expression of the pro-apoptotic protein Bak [104]. Another study, however, observed significantly decreased miR-125b expression in PTX-resistant BC cells. Ectopic expression of miR-125b in MCF-7 and SK-BR-3 PTX-resistant cells sensitized these cells to PTX and reversed the EMT phenotype through regulation of sema4C [105]. Given the discrepancy between the studies, it is clear that the mechanisms of taxane resistance may be dependent on the subtype of BC being treated. Expression of miR-520h confers PTX resistance by protecting the cell from PTX-induced apoptosis through suppression of death-associated protein kinase 2 (DAPK2), a positive regulator of programmed cell death [106]. Induction of DAPK2 expression abrogated miR-520h-induced PTX resistance. PTX treatment of MDA-MB-231 and ZR-75-30 breast cancer cells overexpressing miR-200a resulted in decreased apoptosis. The authors attribute the effects of miR-200a expression to the miRNA’s ability to target TP_53_INP_1_ and YAP1, two proteins involved in the p73-mediated apoptotic pathway [107].

miRs can also be downregulated, leading to resistance. miR-451 and miR-18a-5p are decreased in PTX-resistant BC cells [108,109]. miR-18a-5p regulates CASC2 expression [109], which can lead to PTX resistance by regulating CDK19. CDK19 can also interact directly with miR-18a-5p, although this interaction is novel and not well understood [110]. CASC2 is upregulated in PTX-resistant clinical samples and PTX-resistant MCF-7 and MDA-MB-231 cell lines. Downregulation of CASC2 and CDK19 increased PTX sensitivity in vitro and inhibited tumor growth in vivo [110]. Given this information, the exact mechanism of CASC2-mediated PTX resistance remains unknown but is shown to be actuated by interactions with miR-18a-5p that regulate CDK19 ([110] and reviewed in [111]). Loss of miR-17 and miR-20b confers Taxol resistance in breast cancer by enhancing expression of nuclear receptor coactivator 3 (NCOA3) [112], which is significantly upregulated in Taxol-resistant breast cancer cells. The 3′ untranslated region of NCOA3 contains binding sites for miR-17 and miR-20b, and NCOA3 expression is inhibited upon this interaction. Therefore, this inverse correlation between NCOA3 and miR-17/miR-20b expression may be responsible for taxane resistance in breast cancer.

#### 2.4.2. Long Non-Coding RNAs (lncRNAs)

Long non-coding RNAs (lncRNAs) are easily detectable molecules that make ideal biomarkers when they regulate multiple resistance mechanisms [113]. As a result, some important lncRNAs and their mechanistic pathways will be reviewed to provide a foundation for future research and treatment regimens regarding taxane resistance. Upregulated lncRNA H19 is associated with PTX resistance in ERα-positive BC cells, reducing apoptosis after PTX treatment by preventing transcription of the pro-apoptotic genes *BIK* and *NOXA* [114]. H19 is also upregulated in PTX-resistant TNBC cells, and knockdown of *H19* restored chemosensitivity by repressing the Akt signaling pathway and inducing apoptosis [115]. MA-linc1 is a novel lncRNA regulator of the cell cycle, repressing the expression of its neighboring gene, Purα, whose expression inhibits cell cycle progression. Inhibition of MA-linc1 increases PTX-induced apoptosis, which was rescued by *Purα* knockdown. Further, high levels of MA-linc1 are associated with reduced survival in BC patients. Given that silencing MA-linc1 sensitizes cells to PTX, it may be a potential addition to combination treatment therapies for PTX-resistant tumors ([116] and reviewed in [113]). However, there is still little research surrounding MA-linc1 activity in taxane-resistant breast cancer. The lncRNAs HIF1A-AS2 and AK124454 promote cell proliferation and invasion and also attenuate G2-M arrest in MDA-MB-231, BT549, and Hs578T TNBC cells, contributing to PTX resistance ([117] and reviewed in [118]). While there is likely to be a connection between these lncRNAs and chemoresistance, further research is needed to confirm this association so that lncRNAs HIF1A-AS2 and AK124454 may be used as predictive and prognostic markers for taxane resistance. Long intergenic non-coding RNA, Regulator of Reprogramming (Linc-ROR), induces EMT, which can lead to chemoresistance, through regulation of TGF-β signaling and interaction with miRNAs [119,120,121]. MDA-MB-231 cells overexpressing Linc-ROR had reduced expression of E-cadherin and decreased sensitivity to PTX compared to the shROR cells [122]. Linc-ROR has been proposed as a viable marker for chemoresistance in BC [122,123]. Overexpression of MT-associated protein tau antisense RNA 1 (MAPT)-AS1 reduces the PTX sensitivity of BC cells. In *MAPT-AS1* knockdown cells, apoptotic rates were significantly increased and PTX sensitivity was restored through regulation of MAPT and, subsequently, tau expression [124]. Ferritin heavy chain 1 pseudogene 3 (FTH1P3) is upregulated in PTX-resistant BC tissue and PTX-resistant MCF-7 and MDA-MB-231 cells. FTH1P3 promotes P-gp expression by targeting miR-206 as a miRNA “sponge,” indicating that FTH1P3 may play a role in PTX resistance through regulation of miR-206/ABCB1 ([125] and reviewed in [120]). Overexpression of NONHSAT141924 significantly lowers the survivability of MCF-7 cells by modulating the Bcl-2 apoptosis signaling pathway, suggesting a potential mechanism of resistance. Inhibition of NONHSAT141924 may be an effective strategy to treat PTX-resistant BCs ([126] and reviewed in [127]).

### 2.5. Tumor Suppressor Genes

Tumor suppressor genes play a vital role in response to chemotherapeutic treatment. These genes regulate a variety of biological processes and have loss-of-function in cancers, leading to chemoresistance. Tumor suppressor genes that have been linked to taxane resistance and their underlying mechanisms in BC are further explored below.

#### 2.5.1. Breast Cancer 1 (BRCA1)

BRCA1 is a key regulator of the PTX-induced stress response pathway. BRCA1 aids in the cellular response to anti-microtubule agents by activating the G2-M and spindle assembly checkpoints [128]. BRCA1 interacts with mitogen-activated protein kinase (MAPK) kinase 3 (MEKK3), an upstream regulator of the p38/MAPK and c-Jun NH(2)-terminal kinase/stress-activated protein kinase pathways that is activated upon PTX treatment [129]. In BRCA1 mutant HCC1937 BC cells, BRCA1 was unable to interact with MEKK3, and the response of MEKK3 to PTX treatment was abolished. This reveals that BRCA1 is required for the PTX-induced activation of MEKK3 [129].

#### 2.5.2. Adenomatous Polyposis Coli (APC)

APC is a tumor suppressor that is silenced by mutation or hypermethylation in many sporadic BCs [130]. *APC* knockdown results in PTX resistance in MDA-MB-157 and MDA-MB-231 cells ([131] and unpublished data from our lab). We have shown that the cell cycle proteins, CDK1 and CDK6, are upregulated in the *APC* knockdown cells, which may be responsible for PTX resistance [132]. Another study proposed regulation of APC expression by miR-135. Increased miR-135 was associated with APC downregulation and PTX resistance, although the mechanism of resistance was not determined [133].

#### 2.5.3. p16

Loss of the CDK4/6 endogenous specific inhibitor, p16, can lead to uncontrolled cell proliferation and chemoresistance in TNBC. Low p16 expression may reduce the response of BC cells to chemotherapeutic treatment by inducing cancer stem cell-like properties [134]. Knockdown of p16 in the Rb-inactivated, basal-like breast cancer cell line, BT549, decreased PTX-induced cell death ([135] and reviewed in [134]). However, more studies will be necessary to determine whether p16 expression is an accurate marker for chemotherapeutic response.

#### 2.5.4. Human Expanded (hEx)

The human homolog of *Drosophila* Expanded, human Expanded (hEX), has been shown to possess several tumor suppressor properties in human BCs. hEx drastically decreases cell proliferation in MDA-MB-231 and MDA-MB-436 cells, sensitizing them to Taxol. Much of the Hippo signaling network, which hEx functions within, is conserved from flies to humans [136]. Because the knockdown of both LATS1 and LATS2 Hippo pathway components in hEx-upregulated cells was not able to rescue the hEx phenotype, hEx likely functions independently of the Hippo pathway in these cell lines to inhibit cell proliferation and transformation and mediate drug sensitivity. An alternative mechanism may function whereby hEx prevents S phase progression by upregulating p21 and downregulating cyclin A ([137] and reviewed in [134] and [138]).

#### 2.5.5. Yes-Associated Protein (YAP)

Knockdown of the tumor suppressor YAP in normal breast epithelial cells increased resistance to Taxol-mediated cell cycle arrest, as well as conferring many other cancer phenotypes like increased invasion and migration and inhibition of anoikis ([139] and reviewed in [134]). Many links have been found between YAP and chemoresistance across multiple types of cancers, demonstrating this protein’s viability as a target for improving chemotherapeutic response.

#### 2.5.6. Leucine Zipper Tumor Suppressor 1 (LZTS1)

LZTS1 regulates mitosis by stabilizing MT networks. Decreased LZTS1 expression significantly reduces PTX sensitivity in vitro by decreasing MT stability. In addition, LZTS1-negative tumors were associated with an unfavorable outcome after taxane-based chemotherapy, suggesting that patients’ LZTS1 expression levels may serve as a prognostic factor for BC therapy [140].

### 2.6. Hypoxia Response Pathway

Much like non-coding RNAs, the hypoxia signal response acts mainly by activating or inhibiting specific downstream pathways that control cellular processes that allow for resistance to cytotoxic agents. Upregulation of transcription factor HIF1-α, the most crucial component of the hypoxia pathway, has been frequently observed in breast cancer [49] and is associated with poor prognosis in patients (reviewed in [47]). Moreover, hypoxia has been shown to protect breast cancer cells from taxane treatment-induced apoptosis [141,142]. The hypoxia response can activate many pathways related to resistance in normoxic conditions, so simply upregulating HIF1-α can have many downstream protective effects. For example, hypoxia upregulated anti-apoptotic Mcl-1 and siRNA inhibition of HIF1 rescued the levels of pro-apoptotic proteins Bak, caspase 3, caspase 8, and caspase 10 [142]. Although there is reduced apoptosis and decreased Bcl-2 expression in hypoxic cells, it is not clear if there is a direct protein:protein interaction between HIF1-α and Bcl-2, or if the altered expression comes from a downstream effector, like JNK. Specifically, JNK is activated upon PTX treatment in normoxia and hypoxia but JNK decreases the level of phosphorylated Bcl-2 and Bcl-XL only in hypoxia conditions [141]. In addition, hypoxia enriches the number of BCSCs by upregulating pathways contributing to their “stem-like” proliferation and reduced apoptosis [143,144]. HIF2-α overexpressed breast cancer cells were then monitored for stem cell marker genes (c-Myc, Nanog, and OCT4) with and without PTX treatment, where there was an increase in the stemness genes and increased chemoresistance in cells with HIF2-α overexpression [145]. Hypoxic conditions can also activate autophagy following PTX treatment, which would allow cells to lessen the stress of cytotoxic drugs and prevent total cell death [141]. There is very limited information on the induction of drug efflux pumps by HIF1-α in breast cancer [143], though this seems to connect a possible hypoxia-independent function of HIF1-α, because the upregulation of MDR1 was not observed in hypoxic conditions. This further supports the idea that HIF1-α and the hypoxia response pathway rely on downstream effector pathways to promote survival, which could help to contribute to taxane resistance.

## 3. Taxane Resistance in Ovarian Cancer

Ovarian cancer is the second most common cause of death related to gynecologic malignancies, particularly high-grade serous ovarian cancer (HGSOC) [146]. The front-line treatment consists of cytoreductive surgical resection followed by combination treatment of platinum and taxane drugs. However, more than 70% of ovarian cancer patients relapse after primary therapy within 2–3 years, and almost all recurrent ovarian cancer patients become resistant to chemotherapy, leading to the common causes of death being recurrence and drug resistance [147]. The Multiple Drug Resistance (MDR) phenotype is characterized by decreased intracellular drug concentration, increased expression of drug-metabolizing enzymes, altered cell cycle checkpoint progression, changes in apoptosis or survival pathways, and deregulation of signal transduction pathways. Specific to taxane-treated tumors, abnormal expression of microtubule subunits and associated proteins would directly conflict with the taxane mechanism of action and reduce the bioactivity of the drug. Though research and inhibition of individual pathways shows promise, the sheer number of deregulated or abnormal pathways in taxane-resistant ovarian cancer suggests that a single drug targeting only one will be insufficient. The following is a collection of information from the last decade regarding the proposed mechanisms of taxane resistance in ovarian cancer, highlighting those that have the potential of being therapeutic targets or biomarkers.

### 3.1. Drug Transport and Efflux

The ATP binding cassette transporters (ABC transporters) are a family of efflux pumps that reduce intracellular drug concentrations, allowing cancer cells to develop chemoresistance. P-gp, the transmembrane protein that is encoded by the MDR1/ABCB1 gene, actively participates in drug efflux, with varied substrate specificity [148]. MDR1 expression has been negatively correlated with response to paclitaxel and doxorubicin in multiple cancers [149,150,151] including HGSOC [152,153,154]. However, it is not clear whether the resistance comes from intrinsic overexpression of ABCB1/P-gp, adaptive overexpression in response to PTX treatment [155], or some degree of both. This implies that the primary and second-line treatment could be increasing the protein responsible for resistance and warns clinicians to prescribe alternatives that are not MDR1 substrates.

Regardless of the means of MDR1 expression alteration, the question becomes, “how specifically does MDR1 expression and P-gp activity increase?” It could be that the increase comes from a copy number variation (CNV) in ABCB1/MDR1 following PTX treatment or already present in the cell [156]. Another possibility is a single nucleotide polymorphism (SNP) in MDR1 affecting the expression level and activity and thus influencing the rate of substrate efflux. The impact is questionable due to the already variable nature of the gene and multiple SNPs seen in patient samples [157,158,159,160]. Some have argued that the fusion of MDR1 to a strong promoter would increase the expression of MDR1 further by employing neighbor exons [161,162]. This suggests that the alteration to MDR1 would be illustrated only after transcriptome analysis, and any SNPs or CNVs that occur after the fusion would be left “unseen” if the fusion product was not analyzed. The culmination of all three possibilities is the overexpression of MDR1 and drug efflux of PTX and other MDR1 substrates.

There are inhibitors being used preclinically to combat MDR1-induced resistance in ovarian cancer. Some have attempted to inhibit MDR1/ABCB1 directly [156,163] to reverse PTX resistance in ovarian cancer. Although their modes of action are slightly different, docetaxel resistance in ovarian cancer can also be overcome by inhibiting MDR1 [164]. However, allosteric and competitive inhibitors of MDR1 are not the most successful solutions to the intracellular drug efflux problem, especially since targeting MDR1 comes at the price of inhibiting CYP enzymes and increasing toxicity [148,155]. Targeting ATP to limit the activity of MDR1 [165] may also reverse resistance or prevent it from occurring. Understanding which primary and second-line treatments are MDR1 substrates, the conflicting substrates and pharmacokinetics of MDR1 and CYP enzymes, and shared mechanisms of resistance in multiple chemotherapeutic drugs will help to overcome one of the mechanisms of taxane resistance in ovarian cancer.

### 3.2. Drug Metabolism by CYP Enzymes

Along with reduced intracellular drug concentration due to increased drug efflux, cells with increased drug metabolism present another method of developing resistance to taxanes. The cytochrome P450 (CYP) family of enzymes is instrumental in metabolizing xenobiotics, including chemotherapy drugs, and a few of the subtypes are suggested to have a role in taxane metabolism [166]. Increased expression and oxidizing activity of CYP enzymes leads to increased hydroxylated metabolites of multiple taxanes and decreased drug efficacy due to its reduced concentration. CYP2C8 is the main enzyme responsible for PTX metabolism into 6-α-hydroxypaclitaxel [167,168] and it is expressed in ovarian cancer [20]. SNPs of the CYP2C8 gene are associated with altered binding affinity to PTX and reduced enzyme activity, leading to an increase in PTX concentration and cytotoxic side effects [169]. However, because the enzyme is so crucial for the metabolism of other chemicals within the cell, inhibiting it does not seem to be an option. Although not a potential therapeutic target, CYP2C8 overexpression and genetic variants offer a marker for potential adverse responses to taxanes in ovarian cancer.

CYP3A enzymes are also involved in the metabolism of taxanes. Specifically, CYP3A4 is responsible for the conversion of the primary metabolite 6-α-hydroxypaclitaxel to p-3′-hydroxypaclitaxel [170] and converting DTX into inactive metabolites [20]. Increased CYP3A5 genetic variants were associated with increased thrombocytopenia (a cytotoxic side effect of PTX) [169], suggesting that CYP3A5 must be functional for PTX metabolism to occur. However, analyzing the expression of CYP2C8, 3A4, 3A5, and MDR1 in ovarian cancer following taxane treatment showed no single gene correlation to taxane disposition, but it did connect the ratio of CYP3A5 to MDR1 with DTX clearance (reviewed in [20]). Again, there are no inhibitors currently used to reverse the PTX resistance, partly because inhibition would lead to the increased drug toxicity of CYP3A substrates, but the altered expression and genetic variants in the enzymes offer biomarkers for poor response to PTX. The evidence at least indicates that both play a role in the bioavailability of taxanes and the possible adverse reactions seen in ovarian cancer.

CYP1 enzymes’ role in taxane metabolism is not well understood; however, evidence suggests that it is significant enough to target therapeutically to combat resistance. Studies have shown differential expression between the two CYP1 enzymes amongst OC cell lines, suggesting that individuals with differing levels of expression may respond differently to treatments [171]. Their role in the development of taxane resistance is unclear, but CYP1B1 expression is induced by PTX treatment and inhibiting CYP1B1 reversed resistance to PTX, warranting further investigation [172]. Preclinical studies have demonstrated that the anticancer drug Resveratrol inhibits both CYP1A1 and CYP1B1 by controlling their transcription [173,174]. In addition, the analogue of Resveratrol, 3′-hydroxy-3,4,5,4′-tetramethoxystilbene (DMU-212), reduces CYP1A1 and CYP1B1 transcription, protein expression, and enzymatic activity [175,176]. The metabolites are even more active than DMU-212, targeting cells specifically with upregulated CYP1 enzymes [177,178]. DMU-212 efficacy is demonstrated with arrest of the cell cycle, induction of apoptosis, and inhibition of tumor growth observed in ovarian cancer cells [175,179]. Resveratrol and its analogues are not established to combat taxane resistance and their influence on the amount of active PTX or DTX present in the cell is unknown. Prodrugs that require metabolism in order to be activated and is the substrate to one of the CYP enzymes overexpressed or frequently mutated appear to be solid qualifiers for new therapies.

### 3.3. Alteration of Microtubule Regulatory Proteins and Tubulin Isotypes

#### 3.3.1. MAPs and MAPKs

As previously mentioned, PTX’s mechanism of action centers around hyper-stabilizing microtubules (MTs), where the lack of dynamic polymerization/depolymerization halts the cell’s mitotic progression [30]. A number of proteins interact with MTs and offer a therapeutic target to reduce taxane resistance in ovarian cancer. The protein tau is one such microtubule-associated protein (MAP) that competitively binds MTs in the exact same area as PTX, without the antimitotic effect, and has been associated with taxane resistance in multiple cancers, such as ovarian cancer [180,181,182]. Silencing tau resulted in decreased cell proliferation and increased apoptosis, with increased PTX sensitivity [182,183]. Another study utilized samples from patients who were treated with platinum–taxane chemotherapy and found a correlation with tau expression and resistance to PTX [181]. However, it should be noted that tau expression was only correlated with PTX sensitivity following the first-line treatment, and no data were collected prior to treatment. This implies that tau may be a weak predictive biomarker for taxane response, but it could be helpful if patients do not respond well to first-line treatment.

The microtubule-associated protein (MAP) kinase spleen tyrosine kinase (SYK) has also been shown to mediate chemoresistance to PTX in ovarian cancer [184], although the understanding of SYK’s function and action is limited. Small-molecule inhibition of SYK resulted in increased sensitivity to PTX and offers a potential addition to the first-line therapy to increase apoptosis. The mitotic spindle-associated protein UNC-45A yields an interesting inhibitory effect on Taxol when bound to MTs, destabilizing them only in the absence of additional MAPs or the actomyosin system [185,186]. Two pairs of MAP kinases, IKBKB/STK39 and EDN2/TBK1, also have regulatory roles in MT stability, and inhibition via siRNA further increased ovarian cancer cells’ response to PTX [187]. This suggests that targeting the activating kinases responsible for phosphorylating MAPs is a better alternative to targeting all MAPs, especially if a MAPK can activate more than one MAP. Lastly, the kinesin KIF14 acts as a prognostic marker and potential drug target in ovarian cancer. The kinesin’s function outside of cytokinesis is partially known, and its expression has not been shown to correlate with levels of cytokinesis-related markers [188]. However, inhibition has been demonstrated to have an anti-proliferative effect on ovarian cancer cells, which should be explored further. It is clear that MAPs and MAPKs offer plenty of opportunities for therapeutic targets and biomarkers, but they also offer many drawbacks to targeting MT regulatory proteins because there are multiple proteins that could be upregulated in response to one pathway being silenced. Because the inhibitors do not seem to have overlapping effects on all MAPs, researchers must investigate all pathways and substrates involved in MT dynamics to find a sufficient target.

#### 3.3.2. Tubulin Isotypes

βIII-tubulin expression is correlated with poor progression-free survival and chemoresistance to taxanes in ovarian cancer [189]. Moreover, a gross reduction in tubulin polymerization was observed between taxane-resistant and control ovarian cancer cell lines, even following PTX treatment, and βIII-tubulin was found to be overexpressed in a majority of them [190]. The overall reduction of tubulin polymerization demonstrates the ability to overcome the hyper-polymerization induced by taxanes. However, it is even more interesting that the amount of βIII-tubulin expressed was significantly reduced in patients receiving removal surgery prior to taxane treatment compared to patients undergoing treatment prior to surgery [191]. This suggests that tumor cells can be primed by taxanes to upregulate βIII-tubulin expression in order to evade the drug.

This proposes the upregulation of βIII-tubulin occurring in response to the taxane treatment, possibly as a way of mediating resistance. Analyzing the isotype structurally, there is a single Alanine difference between βIII-tubulin and the other isotypes in the binding domain that Taxol targets, which alters the binding affinity between βIII-tubulin and Taxol [35]. The difference in this one amino acid in the binding domain could influence drug resistance via reduced binding to Taxol and subsequent upregulation of βIII-tubulin to combat the suppression of MT dynamic activity. Utilizing peloruside A (PLA), another microtubule-stabilizing non-taxane drug with a different tubulin binding site, to compare mechanisms of resistance between the different tubulin isotypes determined that PLA sensitivity was gained when silencing βII- and βIII-tubulin in PLA-resistant ovarian cancer cells [192]. However, it is noteworthy that the silencing of the isotypes had no effect on PTX sensitivity, indicating that the role of βIII-tubulin is different for the two different drugs or that the drugs themselves activate different apoptotic pathways following MT stability. Comparing βIII-tubulin and βIIa/b-tubulin, the difference in the number of non-taxane binding sites can also directly influence the binding of PLA [193]. Therefore, more research on βIII-tubulin and its interactions should be done in order to better create inhibitory PLA analogs. However, the previous evidence clearly establishes increased βIII-tubulin as a method of overcoming taxane-induced MT stability, as well as utilizing it as a potential biomarker.

### 3.4. Cell Cycle Progression

#### 3.4.1. Cyclin E1 Amplification

Cyclin E1 (CCNE1) complexes with CDK2 to regulate the transition from G1 to S phase, marking the start of DNA replication and the entrance into the cell cycle. With an increase in CCNE1, cancer cells can further manipulate cell replication by amplifying CCNE1, CDK2, and E2F, entering into a positive feedback loop charging through to the S phase [194]. With this increase in DNA synthesis and replication at an uncontrolled rate, the chances of chromosomal errors and instability also increase, leading to mutations of genes related to cell cycle regulation and survival [195]. This CCNE1-dependent upregulation in cell cycle-driving genes and pro-survival genes could then lead to increased cell proliferation in chemo-resistant cells.

It is not known if the acquisition of increased CCNE1 copy number/gene expression occurs early in disease progression or in response to chemotherapeutic treatment. Because the amplification of CCNE1 leads to increased cell proliferation, it could just be an advantageous trait gained by cancer cells to help them survive in general and would then allow cells that develop resistance to chemotherapy to proliferate uninhibited [196,197]. In an effort to combat the influence of amplified CCNE1 on platinum–taxane resistance or HGSOC disease progression, CDK2 inhibitors and siRNA targeting CDK2 have shown to be effective at suppressing the pathway hyper-activated by CCNE1 [198,199]. Interestingly, any resistance observed with the CDK2 inhibitors is believed to be due to the upregulation of CDK2 itself and selection of pre-existing polyploid cells, frequently observed in CCNE1-amplified tumor cell populations [200]. A small-molecule inhibitor for Polo-like kinase 1 (PLK1), a key regulator of mitotic entry and exit, was also used in combination with PTX on HGSOC cells with amplified CCNE1 and showed increased apoptosis [201]. The rationalization comes from the stabilization of ubiquitin ligase component FBW7, which negatively regulates CCNE1 and anti-apoptotic Mcl-1, by PTX and the PLK1 inhibitor BI6727.

The role of CCNE1 in regulating the cell cycle, and its increase associated with poor overall patient survival, demonstrates its ability for use as a potential biomarker for poor prognosis or as a therapeutic target to mitigate resistance in HGSOC. Although there is a connection between CCNE1 amplification and the development of chemoresistance, indirect inhibition of CCNE1 via CDK2 or PLK1 offers a more promising and feasible option.

#### 3.4.2. Cyclin A1

Although not as important as the other A-type cyclin (cyclin A2/cyclin A), cyclin A1 also is involved with the development of taxane resistance in ovarian cancer. Few studies have shown that increased CCNA1 expression is seen in high-grade epithelial ovarian cancers [202,203]. CCNA1 is also increased in PTX-resistant ovarian cell lines and silencing CCNA1 rescued the efficacy of PTX on those cells [204]. Notable interactions between CCNA1 and CDK1 [205] and CCNA1 and CDK2 [206] demonstrate the contribution of CCNA1 in the progression of cell proliferation and tumor growth, but only when involved with the kinases. However, the limited number of studies connecting CCNA1 to PTX resistance in HGSOC and studies utilizing the inhibition of CCNA1 to reverse the effects of resistance mark it with low impact as a potential biomarker for chemoresistance or therapeutic target.

#### 3.4.3. Spindle Assembly Checkpoint

The method of action of PTX focuses on the stability of microtubules and initiating the Spindle Assembly Checkpoint (SAC) to prevent the continuation of mitosis. Because cells that are resistant to PTX overcome the mitotic arrest mediated by the SAC signal, a weakening of the signal or reduction of the effector proteins that carry out the signal could be taking place [207]. Proteins that play a large role in accomplishing the active SAC/mitotic arrest response are BUB1, BUBR1 (BUB1-related protein kinase), and MAD2 (mitotic arrest deficiency 2). The exit from mitosis is most prominently regulated by the relaxation of SAC signal and degradation of cyclin B1 [208]. A decrease in cyclin B expression has been observed in PTX-resistant OC cells, in addition to decreased BUB1, marking the loosening of control which the SAC signal has upon mitotic arrest [209]. BUBR1 and MAD2 directly prevent activation of the Anaphase-Promoting Complex (APC/c) by binding to CDC20 and maintaining a closed confirmation [210]. Using siRNA to silence MAD2, PTX-induced apoptosis was reduced and demonstrated that MAD2 levels play a definite role in the SAC signal, mitotic arrest, and arrest-induced apoptosis [211,212,213]. Moreover, decreased levels of MAD2 have even been hypothesized to be a factor in tumorigenesis in mucinous OC [214], suggesting that MAD2 is more of a driving factor in cell proliferation and disease progression than any other SAC-related protein. All of the above demonstrates the potential use of MAD2 levels as a potential biomarker for at least SAC signal attenuation, but possibly taxane response and prognosis.

#### 3.4.4. Mitotic Exit

Previously, the weakening of the SAC signal and effector proteins were ways that ovarian cancer cells could overcome PTX-induced mitotic arrest. However, manipulating the APC/c and proteins regulating mitotic exit may provide another route to overcome arrest and develop resistance to taxanes. APC/c is an E3 ligase that adds ubiquitin to substrates—most notably, cyclin B1—and targets them for degradation in order to progress the cell cycle through to anaphase [215,216]. In PTX-induced mitotic arrest, the SAC is activated and the APC/c is inhibited by interference of CDC20. When the APC/c is inhibited, cyclin B1 is not degraded, sister chromatids do not separate, and microtubules are theoretically attempting to reconnect properly with kinetochores [217]. However, with the addition of PTX, microtubules are no longer dynamic and cells stay in this “non-mitotic mitosis limbo” until they die or enter senescence [215,218].

However, the majority of the studies attempting to prevent the escape from mitotic arrest emphasized that instead of targeting APC/c to prevent mitotic exit, targeting PLK1 is a better alternative. PLK1 activates the SAC signal when inhibited [216]. PLK1 expression has been connected with disease prognosis and sensitivity to chemotherapy in ovarian cancer, which presents a potential biomarker or therapeutic target in combating resistance [201,216,219,220]. Studies have shown that inhibiting PLK1 in the presence of PTX [201,216] resensitized ovarian cancer cells to PTX and reduced cell proliferation, as well as inducing apoptosis in HGSOC cells with CCNE1 amplification. Targeting the machinery responsible for mitotic slippage and mitotic catastrophe seems to be the best option in combating mitotic cells resistant to anti-mitotic drugs such as taxanes.

### 3.5. Pro-Survival and Anti-Apoptotic Proteins

Assuming that drug concentration is not impacted by the activity of MDR1, phases of the cell cycle are not forced by irregularly expressed cyclins, the SAC signal is not silenced without prerequisites being satisfied, and the APC/c is not prematurely activated, the taxane-treated cancer cell still needs to activate pro-apoptotic pathways and silence anti-apoptotic/pro-survival pathways. Probably the most important question regarding the efficacy of anti-mitotic chemotherapeutic drugs is, “if the cells are no longer mitotically active, what mechanisms lead to their death?”.

#### 3.5.1. BCL-2 Family

The intrinsic apoptotic pathway requires many regulator and effector proteins, and the B cell lymphoma 2 (BCL-2) family comprises a large number of those involved. Although the BCL-2 family has both apoptosis stimulating and inhibiting proteins, overexpression of the pro-survival members (Mcl-1, Bcl-XL, Bcl-2) relates to disease prognosis and response to chemotherapy in HGSOC [221,222,223]. Mcl-1 prevents mitochondrial outer membrane permeabilization (MOMP), the release of pro-apoptotic factors into the cytoplasm, and can dictate the progression of cell death in cancers [224,225]. Mcl-1 ubiquitination and successive degradation activate the intracellular apoptotic pathway [223,226,227,228]. Stabilization of Mcl-1 confers resistance to taxane or platinum chemotherapies [223,226,227] by either blocking the ubiquitin ligase from binding or by deubiquitinating Mcl-1. This evidence suggests that Mcl-1 stability is crucial for ovarian cancers to halt the progression of the cell death pathway, but more research needs to be done to determine the full scope of Mcl-1 protein:protein interactions.

A better strategy may be to target downstream members of the apoptotic pathway with limited binding partners. For example, Bcl-XL and Bcl-2 bind to Bax or Bak (pro-apoptotic proteins) and the sequestering of this heterodimer directly inhibits the permeability of the mitochondrial membrane, the release of cytochrome c, and the continuation of the apoptotic cascade [221,229,230]. An imbalance of Bcl-XL/Bcl-2 to Bax expression can lead to suppressed apoptosis, and overexpression has been associated with chemoresistance in ovarian cancer [153,221,231]. In addition, Bcl-2 genetic variants have been connected to increased resistance to PTX [232]. This evidence clearly points to the involvement of Bcl-2 in mediating resistance to PTX-induced apoptosis. Moreover, an inhibitor to prevent Bcl-XL/Bcl-2 from binding to Bax could provide a solution to the lack of cell death in taxane-resistant ovarian cancer. Utilizing siRNA to knockdown Bcl-XL decreases cell survival and resensitizes to chemotherapy [153,229,233]. In addition, the competitive inhibitor Navitoclax (ABT-263) shows promise in inhibiting Bcl-XL and Bcl-2 by mimicking the BH3 domain of Bad, and multiple studies have illustrated its ability to decrease overall cell survival and reduce chemoresistance in ovarian cancer [229,230,231,233]. By understanding the large role that the BCL-2 family plays in mediating the apoptosis pathway, researchers can therapeutically target irregularities in Mcl-1, Bcl-2, and Bcl-XL expression and activity to combat taxane resistance.

#### 3.5.2. IAP Family

The inhibitors of the apoptosis (IAP) family of proteins are also crucial in regulating cell death and offer potential therapeutic targets. c-IAP1 and XIAP (X-linked IAP) overexpression is associated with chemoresistance and as a biomarker for ovarian cancer [234,235,236,237,238]. Caspase 9, a critical initiator of caspase-mediated apoptosis, is inhibited by the BIR3 domain of XIAP, preventing activation of caspase 9 and subsequent effector caspases in the intrinsic apoptotic pathway [221,229,239]. Inhibition of XIAP, via small-molecule inhibitors or siRNA, increased cell death and reversed chemoresistance [234,236,240]. However, broad IAP inhibitors have shown that the inactivation of c-IAP1/2 will also activate the TNF-α -mediated apoptotic pathway [235,241].

Survivin is another IAP member that actively blocks caspases 3 and 7. Survivin overexpression has been correlated with many cancers, including HGSOC, and reduced sensitivity to taxanes [229,242,243,244]. Silencing survivin via inhibitor, knockout, or adenovirus-mediated knockdown increased apoptosis, reversed drug sensitivity to chemotherapy drugs, and suppressed tumor growth in ovarian cancer [242,243,244,245]. Despite this efficacy, targeting survivin remains difficult due to its normal roles in mitosis and motility. In addition, survivin is not a “conventional” drug target because it interacts with so many other proteins in different pathways [241,246]. With this in mind, the development of a more direct target in the survivin pathway or utilizing other broad IAPs to combat apoptosis suppression in taxane-resistant cells could alleviate these issues.

### 3.6. Signal Transduction Pathways

#### 3.6.1. PI3K/AKT/mTOR Pathway

The phosphoinositol 3 kinase (PI3K)/protein kinase B (AKT)/mammalian target of rapamycin (mTOR) (PI3K/AKT/mTOR) pathway is also a target in overcoming taxane resistance in ovarian cancer. Expression of the PI3K/AKT pathway is often abnormal in cancers, including ovarian, and correlates with reduced overall patient survival and chemoresistance [221,247,248]. Although the PI3K/AKT/mTOR pathway is usually overexpressed, deciphering all of the components and members in the pathway in order to utilize one as a biomarker proves challenging [249,250]. Instead, the use of inhibitors targeting different aspects of the pathway has shown success. Multiple studies have utilized specific inhibitors of the pathway (AZD8835, AZD8186, and D-11688) and silencing RNA to increase the number of apoptotic cells in a dose-dependent manner, reduce anchorage-independent growth, and sensitize resistant cells to chemotherapy [251,252,253]. Interestingly, the combination of a PI3K pathway inhibitor with a BCL-2 family inhibitor showed synergistic effects in increasing cell death [254,255]. It could be that because of the many facets in the PI3K/AKT/mTOR pathway and the fact that the pathway itself has a major role in so many different cellular activities, inhibitors of PI3K/AKT/mTOR should be used in addition to inhibitors of other anti-apoptotic pathways in order to successfully enforce apoptosis in taxane-resistant cells.

#### 3.6.2. Src Family Kinases

Src family kinases (SFKs) are non-receptor tyrosine kinases involved in a number of signaling pathways related to proliferation, induction of apoptosis, and motility and could be used in combination with PTX to aid in efficacy [85,256]. SFKs are overexpressed in cancers, with c-Src being consistently overexpressed in ovarian cancer [257,258]. Evidence of c-Src playing a role in cell proliferation, cell adhesion, metastasis, and angiogenesis characterizes it as a proto-oncogene and suggests a therapeutic target for reducing tumor growth and combating taxane resistance [257]. In addition, Src protein levels and activation were increased after PTX treatment in OC cell lines and ascites of HGSOC patients [259]. Inhibition of SFKs with dasatinib reduces proliferation, induces autophagy and apoptosis in ovarian cancer cells, and reduces tumor growth in mouse models [260,261,262]. Moreover, studies utilizing dasatinib and cytotoxic drugs, such as PTX, have shown synergistic inhibition of proliferation rate [263,264,265]. Another SFK inhibitor, saracatinib, reduces ovarian cancer cell proliferation [266]. SFK inhibition alone has not proven to be more effective at inducing apoptosis or reducing proliferation than PTX, so any drugs targeting SFKs would only prime the cell to cytotoxic effects of PTX. However, because there is evidence that Src levels respond to PTX treatment, Src and SFKs should be investigated further to explore possible involvement in mechanisms of PTX resistance.

### 3.7. Non-Coding RNA

#### 3.7.1. Micro-RNAs

There is a staggering number of miRNAs that are related to cancer, but a much shorter list includes those relevant to OC and mediating taxane resistance. miR-1307 is overexpressed in taxane-resistant OC cells and the inhibition demonstrated a suppression of resistance to PTX while the overexpression promoted chemoresistance [267]. This mechanism is believed to be due to miRNA-mediated suppression of ING5, whose expression promotes proliferation and inhibits apoptosis. miR-433 is similarly overexpressed in PTX-resistant OC cell lines, with four direct target genes that are involved in senescence [268]. Interestingly enough, it is presumed that cells overexpressing miR-433 are encouraging a community transition to senescence by releasing exosomes containing miR-433. Senescence seems like a fair trade-off for chemotherapy-treated cells in that they are no longer active in the cell cycle and their mutations can no longer drive survival; however, cells that emerge from senescence often display resistance to the therapy that elicited this response [269]. Data demonstrate that miR-630 overexpression is connected to PTX-resistant OC and it may play a mechanistic role in mediating the resistance via targeting apoptosis-related proteins [270]. The study illustrates that inhibition increased apoptosis and enhanced sensitivity to PTX, and the combination of inhibitor and PTX produced a synergistic sensitivity to apoptosis induction.

Just as there are miRNAs that mediate taxane resistance through their overexpression, there are a number that have decreased levels in resistant cancer cells, suggesting an inhibitory role on their direct targets. For example, low miR-146 has been observed in epithelial OC, resulting in increased proliferation, decreased apoptosis, and increased sensitivity to taxanes [271]. The mechanism suggests that the low levels of miR-146 allowed for higher levels of SOD2, a protein responsible for converting ROS superoxide into safer hydrogen peroxide, leading to decreased ROS-induced apoptosis. Similarly, downregulation of miR-194 has been connected to increased levels of MDM2, the negative regulator of p53 in PTX-resistant cells [272]. Transfecting PTX-resistant cells with miR-194 increased PTX sensitivity, and transfecting PTX-sensitive cells with anti-miR-194 reduced sensitivity to PTX, hinting at the importance of normal p53 function and the chaos that follows any dysregulation. Decreased miR-133b can influence the expression of its targets, MDR1 and the detoxifying enzyme glutathione s-transferase-π (GSTπ), both of which already correlated with chemoresistance [273,274,275]. This is supported by the low levels of miR-133b observed in chemo-resistant OC cells compared to chemo-sensitive cells, the downregulation upon transfection with miR-133b, and upregulation of MDR1 and GSTπ upon transfection with anti-miR-133b. Interestingly, miR-106a and miR-591 seem to be connected in a mechanism mediating PTX resistance, though their expressions are inversely related [276]. Increased miR-106a is associated with decreased miR-591 in PTX-resistant OC, and inhibiting miR-106a or restoring miR-591 sensitizes PTX resistant cells. Their targets, Bcl-10 and caspase 7 targeted by miR-106a and ZEB1 targeted by miR-591, do not appear to be connected in mechanisms either, which could suggest that they are not truly linked. miR-200c is decreased in PTX-resistant cells and its restoration is associated with increased PTX sensitivity in vitro and decreased tumor formation in vivo [277,278]. However, in one study attempting to restore and suppress levels of miR-200c and miR-141, inhibition through lentiviral transfection produced cells resistant to PTX but the attempted restoration through retroviral particles containing clusters of miR-200 did not restore sensitivity to the drug [278]. This demonstrates that only specific techniques can be used to transport inhibitors or pre-miRNA, which should be considered when attempting to develop a therapy targeting miRNAs.

#### 3.7.2. Long Non-Coding RNAs

Long non-coding RNAs (lncRNAs) are significantly longer than miRNAs and are involved in cellular activities such as chromatin modification, transcription, post-transcriptional modification, and scaffolding [279,280]. lncRNAs can maintain RNA–protein interactions, allowing them to recruit complexes and act as regulators [281]. For instance, FER1L4 (lncRNA Fer-1-like protein 4) downregulation led to increased PTX resistance and directly influenced activation of mitogen-associated protein kinase (MAPK), which plays a role in cell proliferation, differentiation, and cell death [282,283]. Phosphorylation and activation of MAPK and ERK was observed in PTX-resistant cells, where FER1L4 was low, and transfection of FER1L4 decreased MAPK/ERK phosphorylation. However, it is also possible for lncRNAs to play a role in taxane resistance by utilizing miRNA intermediates. Such is the case with UCA1, where knockdown negatively regulated miR-129 and ABCB1/MDR1 to sensitize cells to PTX [284]. Similarly, LINC01118 siRNA overexpression suppressed PTX sensitivity and reduced the inhibitory rates lower than the PTX-treated control by inhibiting miR-134 and thus upregulating ABCC1/MRP1 [285]. Lastly, NEAT1 inhibits miR-194 to regulate expression of ZEB1, which is associated with the acquisition of cancer stem cell-like properties and the promotion of chemoresistance [286,287]. Overall, it is clear that miRNAs and lncRNAs have well-documented involvement in developing taxane resistance in ovarian cancer; however, since the list is so lengthy, targeting only one would prove inefficient and their expression might be better utilized as a biomarker for taxane response.

### 3.8. Hypoxia Response Pathway

Although upregulation of hypoxia response transcription factor HIF1-α is frequently observed in ovarian cancer, an established relationship between hypoxia and resistance to taxane chemotherapy has yet to be elucidated [49,288]. Interactions between HIF1-α and multiple resistance pathways and proteins suggest that hypoxia plays a role in mediating reduced apoptosis following taxane treatment. For example, the stability and downstream transcription factor activity of HIF1-α is mediated by c-Src in hypoxic conditions and leads to chemoresistance [289]. More specifically, genetic and protein inhibition of c-Src restores PTX sensitivity and decreases HIF1-α levels [290]. Upregulated miR-21 presents a possible relationship between MDR1 and HIF1-α, where siRNA inhibition of miR-21 decreased both MDR1 and HIF1-α expression and restored sensitivity to PTX [291]. Moreover, siRNA inhibition of HIF1-α also decreased MDR1 and restored PTX sensitivity [291]. MDR1 expression was increased in hypoxic conditions but was reduced following treatment with the clinical topoisomerase 1 inhibitor topotecan (TPT) [292], further supporting the interaction between HIF1-α levels and drug efflux pumps in taxane resistance. TPT indirectly inhibits HIF1-α by preventing its translation [292,293]. Though successful at inhibiting HIF1-α, TPT is not effective enough as a monotherapy and may only benefit patients with increased hypoxia signal and the presence of topoisomerase 1. In fact, it was found that TPT should be used as a second-line therapy due to the efficacy not being markedly better than PTX [294]. HIF1-α was also found to interact with p53 in hypoxic conditions using co-IP, possibly due to inhibition by HIF1-α on p53’s pro-apoptotic function [292]. It is clear that HIF1-α coordinates with multiple resistance proteins in ovarian cancer, but more research should be done on the prevalence of hypoxia in ovarian solid tumors before assigning HIF1-α to biomarker status. In addition, targeting downstream affected pathways may be a better alternative to HIF1-α specific inhibitors because targeting HIF1-α in healthy cells may prove detrimental.

## 4. Taxane Resistance in Prostate Cancer

Prostate cancer is the second most common form of cancer in men and the second leading cause of cancer-related death in men in the US [295]. The development of castration-resistant prostate cancer (CRPC) in response to first-line treatment androgen deprivation therapy (ADT) frequently occurs in patients, which leads to disease progression and use of taxane chemotherapy [296]. DTX is the primary drug used in the treatment of CRPC, but CBZ, the newest generation of taxanes, has been utilized as a second-line treatment for CRPC when DTX resistance inevitably occurs [37]. Though CBZ is initially effective, CRPC eventually stops responding to the drug and tumor progression continues until the patient succumbs to the disease [297]. Combinations of new anti-androgens that compete for AR active sites (enzalutamide and bicalutamide) or inhibit synthesis of androgens (abiraterone) are still being tested for clinical efficacy with ADT and taxanes as a third-line treatment to combat resistance or as a modified first-line to avoid resistance altogether [298,299,300,301,302]. The exact causes of DTX and CBZ resistance are still being investigated, but popular mechanisms of drug resistance appear to play a role. These include the following: decreased intracellular drug concentration mediated by ATP-binding pumps; altered microtubule dynamics and roles in signaling pathways; transition to stem cell-like phenotype; upregulation of pro-survival pathways; downregulation of pro-apoptotic pathways; and dysregulation of non-coding RNA expression [303,304,305]. While these pathways have been observed in other cancer types, the following is a summary from the past decade describing those pathways in CRPC-related taxane resistance, as well as the other pathways that are unique to prostate cancer.

### 4.1. Intracellular Drug Concentration and Transport

#### 4.1.1. MDR1

As demonstrated with other taxane-treated cancers, the substrate-binding ability of MDR1 to taxanes such as PTX and DTX is also seen in CRPC, and the increased efflux of the drug reduces its efficacy within the cell [37,303]. Increased MDR1 expression has been identified in DTX-resistant CRPC cells, and use of siRNA or specific inhibitors to knockdown activity of MDR1 has restored DTX sensitivity [306,307,308]. Interestingly, the basal levels of MDR1 have varied across cell lines and are associated with differing responses to DTX following MDR1 inhibition/knockdown [309]. This suggests that the cell lines with lower/no expression of MDR1 could mediate resistance to DTX via other pathways or other transporter proteins [310].

The specific upstream pathways that induce MDR1 in CRPC have yet to be elucidated. Some have demonstrated that the upregulation comes from hyperactivity of the pro-survival and cell proliferation pathway, PI3K/AKT, mediated by epidermal growth factor receptor (EGFR) [311,312]. The EGFR pathway activates pathways involved in cell proliferation and migration [313], and inhibition of EGFR decreased MDR1 expression and increased DTX sensitivity in DTX-resistant cells [311,314]. The transcription factor E26 transformation sequence 1 (ETS1) also has a role in MDR1 upregulation and taxane resistance, which is supported by the increased sensitivity to PTX and decreased MDR1 mRNA and protein levels observed after knocking down ETS1 [315]. Inhibition of the nuclear receptor RORγ also led to a reduction in MDR1 expression and protein levels [316]. This inhibition then restored response to DTX and CBZ in cross-resistant CRPC cells. Interestingly, extracellular signals can also lead to resistance phenotype, as demonstrated when DTX resistance was conferred to naive cells via exosomes containing MDR1 [317]. Exosomes isolated from serum from patients not responsive to DTX contained MDR1 and conferred DTX resistance to DTX-sensitive cells [318]. This suggests that only a few CRPC cells that respond to taxane treatment with upregulation of MDR1 are necessary to influence the rest of the cell population and further develop resistance. Whether the increase comes from intracellular pathways, transcription factors, or extracellular vesicles, the upregulation of MDR1 results in taxane resistance.

However, inhibiting MDR1 directly may not be the method to overcome drug efflux and resistance to taxanes. Despite positive in vitro and in vivo studies [163,306,319,320,321], clinically, MDR1 inhibitors have not been successful. This could be due to the increased toxicity of taxanes, increased levels of supplementary drug transporters, or upregulation of other resistance mechanisms. Use of anti-androgens, such as bicalutamide, that block androgen receptor signaling have been observed to decrease MDR1-mediated DTX efflux and restore sensitivity to DTX-resistant CRPC in vitro and in vivo [322]. The development of CBZ as a third-generation taxane was hoped to overcome DTX resistance and disease progression in CRPC, and clinical data demonstrated that CBZ was still effective in DTX-resistant patients [297,323]. Because CBZ is not a substrate of MDR1, this would eliminate one of the most prominent methods of resistance to taxanes [19]. However, some studies connect CBZ resistance with an increase in ABCB1 expression and MDR1 protein levels in CRPC cells [307,324,325]. Another study demonstrates that inhibition of MDR1 re-sensitizes resistant cells to CBZ in DTX/CBZ cross-resistant PC3 cells, suggesting that MDR1 does play a role in CBZ resistance [324]. Due to opposing evidence that there is no correlation with MDR1 and CBZ response in taxane-resistant cells [42,324], CBZ sensitivity could depend on basal expression of MDR1 and the levels of other drug efflux pumps. Therefore, utilizing MDR1 and other ABC transporter proteins as biomarkers for taxane response could be more useful than targeting the proteins directly.

#### 4.1.2. SLCO Genes and Drug Influx

Another mechanism of reducing intracellular taxane concentration and promoting resistance is to reduce the expression or activity of drug influx transporters. Evidence connects single nucleotide polymorphisms (SNPs) and loss of activity in solute carrier of organic anions (SLCO) genes with reduced response to taxanes in CRPC patients [326,327,328]. Furthermore, there are higher amounts of DTX in plasma than in the liver (where it would normally be metabolized) in Slco1b2 knockout mice ([329], reviewed in [330]); this suggests that SLCO proteins (Slco in mice) are important for transporting DTX into cells, and loss could lead to reduced intracellular DTX concentrations. In vivo studies demonstrated that genetic variations of SLCO genes corresponded to impaired DTX influx [331], meaning that polymorphisms and expression levels could be potential indicators for taxane uptake or resistance. In fact, *SLCO1B3* was one of the most downregulated genes in DTX-resistant prostate cell lines and overexpression led to increased taxane sensitivity [332]. Patient-derived xenograft models of CRPC correlated decreased intratumoral concentrations of DTX with increased resistance to DTX, further strengthening the role of influx and efflux as potential mechanisms for taxane resistance [333]. Both increased drug efflux and drug influx create issues with accurately dosing a patient with high enough taxane concentration, where increasing doses could only increase the cytotoxic effects from taxanes. SLCO genes that operate also as efflux pumps could also be partaking in the removal of taxanes prior to action [334]. More research should be done on the impact of SLCO influx transporters in CRPC and to determine if there is a possible mechanism of resistance from decreased taxane uptake. In addition, levels of SLCO gene expression and mutations may act as biomarkers for altered drug response.

### 4.2. Microtubule Dynamics and AR Signaling Pathway

#### 4.2.1. β-Tubulin Isotypes and Mutations

Besides maintaining intracellular concentrations, the efficiency of taxanes in cancer cells also relies on their interaction with their intracellular targets, microtubules (MTs), where changes in tubulin composition could alter taxane binding and influence drug sensitivity. A mutation in one of the isotypes of tubulin, class I β-tubulin, could reduce binding to taxanes [335]; however, there is no clinical correlation to drug sensitivity and resistance. βIII-tubulin binds significantly less to taxanes than the other forms of tubulin due to a single amino acid difference [35]. Cells in multiple cancer types, including CRPC, with overexpression of βIII-tubulin have reduced sensitivity to taxane drugs [31,37,336]. Analysis of βIII-tubulin expression in a wide range of tumors from prostate cancer patients demonstrated that high expression of βIII-tubulin correlated with reduced response to DTX [337]. The study by Ploussard et al. even suggested that the increase in βIII-tubulin expression could be a result of taxane treatment after analyzing samples from patients with/without treatment as well as those sensitive/resistant to treatment [337]. More evidence has connected higher levels of βIII-tubulin in tissue samples from CRPC patients having at least three cycles of DTX with shorter overall survival and reduced responsiveness to taxane treatment [338]. βIII-tubulin levels are strongly associated with taxane resistance in CRPC and could act as a biomarker to predict patient response prior to treatment, enabling clinicians to prescribe alternatives and avoid resistance.

In addition, in response to taxane treatment, βIII-tubulin is upregulated, which could highlight specific cellular pathways leading directly to resistance and offer therapeutic targets. Some have suggested that cancer cells recognize a stress signal following taxane treatment and respond with upregulating isotypes of tubulin that allow them to increase MT dynamics, upregulate survival signaling pathways, or evade forced apoptosis [339]. One study quantified the amount of polymerized and stable MTs that self-arrange in clumps or bundles, known as MT bundling, that occurs in taxane-sensitive or taxane-resistant tumors following treatment [340,341]. The amount of bundling was then correlated with the documented responsiveness of the patient to therapy, where decreased bundling was observed in patients that had recorded resistance to therapy. This suggests that the binding of taxane drugs with MTs is the first marker of treatment sensitivity and also the first point where resistance can occur.

Because of the clear role in taxane efficacy and patient response, targeting the individual isotypes that mediate resistance could be helpful. The silencing of *TUBB3*, the gene that encodes for βIII-tubulin, did not interfere with the other isotypes of tubulin and restored sensitivity to DTX in DTX-resistant prostate cancer cell lines, while the overexpression conferred resistance to previously sensitive cells [337]. Even further, *TUBB3* knockdown restored sensitivity to CBZ in CBZ-resistant cell lines and increased the sensitivity of CBZ-resistant cells to DTX [342], indicating that MT composition and their binding to taxanes could be a shared mechanism in DTX and CBZ resistance. However, other research claims that CBZ demonstrates better MT dynamics suppression when βIII-tubulin levels are high (or are at normal levels compared to depleted levels) [343], suggesting that CBZ may bind better to this isotype than other taxanes [344]. It should be noted that just because CBZ binds better to βIII-tubulin, it does not only bind to βIII-tubulin, meaning that CBZ efficacy is not completely inhibited when *TUBB3* is knocked down. Moreover, if tubulin isotypes are upregulated and taxane concentrations are already reduced, as discussed in earlier sections, then MT depolymerization and resistance could occur. The role of MT isotypes and the genes that encode for them, in mechanisms of resistance, is not fully understood and more research should be done in exploring small-molecule inhibitors of *TUBB3* or possible regulators that control βIII-tubulin expression and overexpression in taxane-resistant CRPC.

#### 4.2.2. AR Signaling

Aside from making up the mitotic spindle, MTs also play an important role in the localization of androgen receptor (AR) to the nucleus. AR is a ligand-dependent nuclear transcription factor that activates genes essential for proliferation and differentiation [345] and is necessary for prostate cancer progression [346,347]. When AR binds to androgens in the cytoplasm, MTs aid in localization to the nucleus, creating an opportunity for signaling inhibition when MTs are stabilized by taxanes. In fact, studies have demonstrated that cells derived from DTX-treated CRPC patients lack AR nuclear localization and that MTs directly bind to AR [348,349,350]. There is evidence that the amount of nuclear localization of AR negatively correlates with clinical responsiveness to taxane treatment, where cytoplasmic sequestration of AR is associated with increased MT bundling and increased response to taxanes [341,351]. Likewise, there is a connection between DTX resistance and increased AR signaling [335], indicating an association between taxane efficacy and the AR signaling pathway mediated by MTs.

However, there is much debate about whether aberrant AR signaling causes taxane resistance and how exactly this is accomplished. Once AR is localized to the nucleus, AR and AR-mediated transcription is regulated by Lysine-specific demethylase 5D (KDM5D), and KDM5D knockdown increases AR signaling and confers DTX resistance to cells [352]. The splicing of AR into variants has been indicated to avoid AR inhibition via taxanes, by altering tubulin binding [353,354]. Interestingly, a reversion from a positive AR variant 7 (ARv7) detection to a negative detection and a conversion from negative to positive ARv7 detection have been observed in DTX- and CBZ-treated patients, indicating that there may be an ease of AR signaling or a ramping up in taxane treatment [355]. However, there is also research that indicates that there is no connection between splice variants and taxane response [355,356]; specifically, knocking down AR variants did not resensitize cells to taxanes [357]. Some have claimed that mutations in AR could influence binding to tubulin in such a way to evade taxane-induced MT stability, but these mutations are rare in CRPC patients [345,358]. Mechanisms of developing resistance to ADT could spill over into taxane resistance if MTs provide the link between the two. For example, the development of fusion proteins with ETS-related gene (ERG) links ETS transcription factors (regulating proliferation, death, and developmental genes) and AR signaling [359]. ERG fusion proteins detected in patient blood and tissue were correlated with reduced prostate-specific antigen (PSA) progression-free survival following DTX or CBZ treatment [360]. However, ERG fusion proteins were actually observed to inhibit AR signaling and serve a regulatory role for the pathway [361,362]. More research should be done on potential taxane cross-resistance following ADT and potentially treating combinatorically instead of sequentially.

Adding specific anti-androgen inhibitors to taxane treatment may combat the influence of AR signaling on resistance. Specifically, enzalutamide is an FDA-approved nonsteroidal AR inhibitor that blocks the binding of AR to androgens in the cytoplasm and has been used alone, with ADT, with taxanes, or with the CYP17A inhibitor abiraterone [298,363,364,365,366]. Preliminary research showed that enzalutamide following taxane treatment increases progression-free survival and clinical trials are investigating the effects of DTX and enzalutamide combination therapy [363,364,367,368]. Interestingly, a significant decrease in MDR1 activity was observed with enzalutamide treatment, to the point where DTX-resistant cells were re-sensitized to DTX [322], suggesting an interaction between AR and efflux pumps that could prove useful for taxane-resistant cells. In vitro studies have demonstrated cross-resistance to taxanes in established enzalutamide-resistant CRPC cells [369], hinting at mechanisms of resistance to both drugs with reactivation of AR signaling. However, other studies have observed no resistance to CBZ in enzalutamide-resistant cells, making it a better suited chemotherapeutic option for cancers resistant to ADT or AR-targeting drugs [370,371,372,373]. Furthermore, clinical studies testing the combination of CBZ with abiraterone demonstrated an increase in PSA, PFS, and antitumor activity in patients previously treated with DTX and abiraterone [365]. The efficacy of anti-androgens on their own is not well established, but upcoming clinical studies may open doors for potential combination therapies to combat resistance in CRPC.

### 4.3. EMT Phenotype

CRPC cells post-ADT or during taxane treatment could develop resistance through EMT. The EMT phenotype plays a role in helping to achieve tumor aggressiveness and drug resistance by upregulating proliferation and pro-survival pathways, both considered stem cell-like characteristics, and generates qualities such as resistance to apoptosis and transdifferentiation [374,375]. EMT is associated with increased metastasis and tumor invasiveness in many cancers and has recently been connected with the development of taxane resistance in CRPC [37,376,377,378]. Analyzing a tumor microarray of prostate cancer samples either untreated or treated with DTX demonstrated decreased E-cadherin, an indicator of EMT, in DTX-treated patients [379]. Furthermore, patient tumor samples also expressed high CD44 populations (an indicator of cell stemness) and had increased vimentin and zinc-finger e-box binding homeobox 1 (ZEB1) expression levels (indicators of EMT) in DTX+ADT-treated patients (who eventually relapsed) compared to untreated patients [378,379]. In vitro studies have observed EMT markers (increased ZEB1 and vimentin, reduced E-cadherin) in DTX-resistant CRPC cell lines [379]. Likewise, ZEB1 knockdown increased cell sensitivity to DTX and decreased CD44 populations, strengthening the argument that EMT can lead to DTX resistance and stem cell-like properties via ZEB1 [377,380].

Transforming growth factor β (TGFβ) has two counteracting roles in inhibiting proliferation/inducing apoptosis and activating EMT/overexpressing anti-apoptotic pathways [374,376,381]. When the pathway is malfunctioning or hijacked in resistant CRPC cells, EMT is induced and resistance to taxane-induced apoptosis can occur. High expression of macrophage inhibitory cytokine 1 (MIC-1), a member of the TGFβ protein family, is correlated with EMT and DTX resistance [382]. Targeting the TGFβ family, demonstrated in vitro by knocking down MIC-1 [382] and in vivo by targeting the ligands in the signaling pathway [383] with specific inhibitors, increased treatment response and offered a new combination therapy to combat taxane resistance. In addition, because AR and EMT modulators have an inverse relationship, treatment with taxanes prior to ADT could mitigate EMT and circumvent resistance pathways [374,379,384]. However, more importantly, studies have shown that EMT and the reverse, mesenchymal-to-epithelial transition (MET), are cyclical and can be induced in both directions [374,381]. Specifically, CBZ and enzalutamide combination treatments appeared to reverse EMT to MET phenotype and reverse resistance [385]. More research needs to be done on understanding at what point EMT occurs, what specifically mediates the transition, and the impact that EMT has on taxane sensitivity. Combinations of existing and approved drugs may reconcile the interaction between EMT and response to taxanes.

### 4.4. Pro-Survival and Anti-Apoptotic Pathways

#### 4.4.1. BCL-2 Protein Family

Assuming that the microtubule inhibition and cell cycle arrest can take place following taxane treatment, CRPC cells still have to submit to apoptotic pathways in order for them to be sensitive to the treatment; without pro-apoptotic pathways and through the upregulation of pro-survival proteins, CRPC cells can become resistant to chemotherapeutics. Specifically, the BCL-2 family contains pro-apoptotic factors, Bad and Bax, and anti-apoptotic factors, Bcl-2, Bcl-XL, and Mcl-1, where upstream pathways activate or inhibit one or the other depending on the situation [386]. The BCL-2 family has the potential to be a therapeutic target; however, it is unclear whether specific family members should be inhibited or if broad-scale inhibitors would be more successful. BH3 mimetics are small-molecule inhibitors that bind to the BH3 domain of BCL-2 family proteins, making them inactive [387]. Different mimetics exist, targeting specific members of the family; for example, ABT-199 targets Bcl-2 specifically, ABT-737 acts more broadly inhibiting Bcl-2 and Bcl-XL, and ABT-263 acts against Bcl-2, Bcl-XL, and Bcl-w [387,388]. Previous attempts to specifically target Bcl-2 alone demonstrated limited success due to the upregulation of Mcl-1 and Bcl-XL to counter the inhibition [389], calling for broad inhibitors that include more BCL-2 members. For example, using small-molecule BCL-2 family inhibitors ABT-263 or ABT-737 can enhance the antitumor activity and cytotoxic effect of DTX on DTX-resistant cell lines more so than ABT-199 because the former can inhibit Bcl-2, Bcl-w, and Bcl-XL [388].

Bcl-2 and Bcl-XL levels are increased in CRPC [390] and have been associated with taxane sensitivity [386,387,388,389]. siRNA targeting of Bcl-2 increased sensitivity in PTX-resistant cells but the cells also contained mutations in PTEN, a negative regulator of the pro-survival PI3K/Akt pathway [391], which could indicate that changes in BCL-2 family expression can influence the expression of other apoptosis-related pathways that contribute to resistance. An increase in apoptosis in vitro and in vivo was observed with Mcl-1 siRNA, knockout, or small-molecule inhibition and ABT-263 combination compared to ABT-199 combination, suggesting that targeting Mcl-1 and Bcl-2 is not enough to increase apoptosis [392]. Furthermore, ABT-263 induced apoptosis in CRPC cells compared to ABT-199, and the combination of ABT-263 with PTX resulted in a synergistic increase in apoptosis and caspase activation [393]. This suggests that Bcl-XL overexpression is crucial enough to pro-survival that inhibiting it could restore taxane sensitivity. However, this response with combination treatment could be due to differing baseline levels of BCL-2 family members in different cell lines; the expression levels of Bcl-2 were far greater in PC3 cells than in LNCaP cells, which could indicate why the amount of apoptosis was increased in the LNCaP cells following Bcl-XL knockdown or inhibition [393,394]. To combat contradicting expression patterns among the BCL-2 family members, targeting upstream regulators may be more effective. For example, siRNA inhibition of NOTCH1 led to decreased proliferation and increased apoptosis in CRPC cells, as well as a reduction in Bcl-2 and increase in pro-apoptotic protein Bax [395]. More research should be done on the upstream genes controlling BCL-2 family expression in order to successfully target all pro-survival members and restore taxane sensitivity.

Many preclinical studies have demonstrated that targeting BCL-2 members is successful at restoring sensitivity to DTX but also relies on proteins involved in cell cycle arrest to increase apoptosis. For example, the efficacy of DTX has been correlated with the ability of CDK1/cyclin B1 to phosphorylate and inactivate Bcl-2 and Bcl-XL, and the cell needs Bax and the CDK1/cyclin B1 complex in order to activate caspase-dependent apoptosis [396]. Because the complex partially phosphorylates Bcl-2 and Bcl-XL in normal mitosis, it has been shown that prolonged mitotic arrest can also inactivate the pro-survival proteins. However, resistant cells could override the arrest; therefore, the reduction in apoptosis seen in taxane-resistant cells could be due to a lack of active CDK1 and activation of BCL-2 family members [392,397]. CDK1 inhibitors roscovitine, purvalanol A, and RO-3306 led to dephosphorylation of Bcl-2/Bcl-XL [398], suggesting that the complex is essential for regulating pro-survival pathways. Although both cell cycle proteins and pro-survival proteins play a role in cell response to taxane-induced apoptosis, no significant increase has been clinically reported yet with DTX and BCL-2 family inhibitors [399]. Due to the upregulation of BCL-2 family members following taxane treatment and the efficacy of BCL-2 family inhibitors only when pro-survival proteins are upregulated, further research and testing should be done on combining available BCL-2 family inhibitors with DTX or CBZ in taxane-resistant CRPC.

#### 4.4.2. PI3K/Akt Pathway

Resistance can also develop from the irregular upregulation of the pro-survival PI3K/Akt pathway through increased expression of the upstream activating kinases or loss of the inhibitory regulators for the two signaling cascades. Irregular expression of PI3K/Akt has been associated with prostate cancer progression but could also play a role in the development of resistance and continuation of the disease during taxane treatment [400,401,402,403,404]].

Loss or inactivation of the negative regulator for PI3K/Akt, phosphatase and tensin homologue deleted on chromosome 10 (PTEN), mutations in PI3K itself, and hyperactivation of Akt have been connected with chemoresistance in CRPC [405,406,407]. For instance, CRPC cells resistant to DTX were treated with the bioactive dietary flavonoid quercetin and DTX, which increased apoptosis and sensitivity to DTX by reducing the expression of phosphorylated Akt [402,408]. In addition, in vitro studies demonstrated that while overexpression of PTEN sensitized cells to PTX, the knockdown of PTEN led to increased resistance to PTX [407]. CRPC cells cultured in an ADT-like medium were more resistant to DTX than their normally cultured parental cells and the resistance also came with an increase in phosphorylated Akt [409]. The activity of phosphorylated Akt increased in response to DTX in a dose-dependent manner, indicating that DTX had a role in upregulating the very mechanism contributing to its resistance. Similarly, the inflammatory chemokine CCL2 was found to be upregulated in prostate cancer patients treated with DTX and in in vitro models of prostate cancer treated with DTX [410]. In both studies, treatment with a PI3K/Akt inhibitor (LY294002) restored sensitivity to DTX and increased apoptosis. In two different methods, one being stimulated by increased AR signaling and the other being induced by inflammatory cytokines in response to taxane treatment, the PI3K/Akt pathway and hyperactivation of the pathway lead to resistance in prostate cancer. Mammalian target of rapamycin (mTOR), a member of the PI3K-related kinase (PIKK) family, can be inhibited by NVP-BEZ235, in addition to PI3K itself. Blocked mTOR leads to decreased phosphorylated Akt levels, increased DTX-induced apoptosis in vitro, and inhibited tumor growth with DTX treatment in vivo [411]. Because use of mTOR-specific inhibitors has insufficient suppression and combination with DTX does not appear to be better at antitumor capabilities than DTX alone [412], the more broad NVP-BEZ235 appears to be the best option for reducing PI3K/Akt signaling and the effects of the pathway on taxane resistance. However, more clinical trials should be done to determine the combined effects of other current therapies and how inhibition of PI3K/Akt may lead to changes in other resistance mechanisms.

### 4.5. Non-Coding RNAs

Alterations in miRNAs/lncRNAs are associated with prostate cancer [413,414]. As the mechanisms behind taxane resistance in CRPC become more defined, the roles of both miRNAs and lncRNAs are emerging [305,415,416,417,418].

#### 4.5.1. miRNAs

Many miRNAs are often downregulated in drug-resistant cell types but under normal cellular conditions help to regulate signaling pathways geared toward pro-survival or inhibited apoptosis. For example, miR-143 targets KRAS, involved in the activation of the oncogenic MAPK/Ras pathway, and its overexpression can sensitize cells to DTX [419]. miR-34a normally inhibits NOTCH (involved in the BCL-2 family pro-survival pathway), but downregulation resulted in PTX resistance in in vitro models [420]. miR-148 also restored sensitivity to PTX by transfecting ectopic miR-148 into resistant cells [305,413,417]. Interestingly, the regulation of E-cadherin and ZEB1 has been correlated with expression of members of the miR200 family; the reduction of the miR-200 members allows for increased ZEB1 expression, which negatively regulates E-cadherin [379,421]. Transfection of miR-200 members into CRPC cells increased E-cadherin and increased DTX-induced apoptosis [379]. Therefore, there is a necessity for novel inhibitors of EMT, either by restoring levels of miR-200, targeting the downstream pathway responsible for the loss of miR-200, or targeting EMT pathway effectors.

Similarly, upregulated miRNAs that are at low levels normally can overstimulate pro-survival pathways or inhibit pro-apoptotic factors. The miR-17 family (specifically miR-21) inhibits programmed cell death 4 (PDCD4) and this relationship leads to the resistant phenotype [422]. Interestingly, inhibition of miR-21 using antisense oligonucleotides (ASO) increases PDCD4 and restores sensitivity to DTX [422]. Given that resistance is likely a combination of multiple mechanisms, inhibiting one would most likely not establish prolonged sensitivity to taxanes. However, they could be utilized as predictive biomarkers for upregulated pathways or mechanisms that have demonstrated correlation with taxane resistance. miRNA mimics have been identified and established from a genome-wide screen hoping to discover miRNAs that could improve sensitivity to DTX or CBZ [415]. Either utilizing these mimics to restore baseline levels and regulation or inhibiting the genes the mimics target could be clinically useful. Research on miRNA mimics and possible inhibitors needs to be done in CRPC patients to test the efficacy and safety but could be a novel therapy to mitigate taxane resistance from miRNA alteration.

#### 4.5.2. lncRNAs

Specific lncRNAs can act as competing endogenous RNAs (ceRNA) that sequester or inhibit the miRNAs involved in pro-apoptotic pathways [423]. The upregulation of key lncRNAs, and subsequent downregulation of corresponding miRNAs, has been associated with resistance to taxanes in CRPC [424,425]. For example, high expression of the lncRNA nuclear paraspeckle assembly transcript 1 (NEAT1) has been connected with reduced expression of miR-34a and miR-204-5p in CRPC patient tumor samples, prostate cancer cells, and DTX-resistant prostate cancer cells [426]. Knockdown of NEAT1 increased miR-34a/miR-204-5p levels and sensitized cells to DTX in vitro. In vivo, NEAT1 knockdown combined with DTX was more effective at reducing tumor growth than DTX alone [426]. This suggests that NEAT1 suppresses DTX-induced cell death and promotes drug resistance through acting as a ceRNA to impede the pro-apoptotic effects of miR-34a and miR-204-5p [427,428]. Similarly, the overexpression of long intergenic non-protein-coding RNA 00518 (linc00518) and subsequent downregulation of miR-216b-5p were observed in CRPC cells with acquired PTX resistance as well as PTX-resistant patient tumors [429]. Knockdown of linc00518 increased miR-216b-5p and sensitized resistant cells to PTX, most likely due to the established interactions between miR-216b-5p and cyclin B1, phosphorylated-Bad, and Bcl-XL [429,430]. Expression of the lncRNA small nucleolar RNA host gene 6 (SNHG6) is associated with decreased miR-186 in PTX-resistant prostate cancer cells and patient tissues [431]. While the knockdown of SNHG6 sensitized resistant cells to PTX and increased miR-186, integrating miR-186 ASO in the SNHG6 knockdown cells reversed the sensitivity and promoted resistance [431]. This demonstrated that SNHG6, like most of the lncRNAs associated with specific miRNAs, acts as a sponge to block the pro-apoptotic activity of miR-186 and avoid drug-induced apoptosis [432]. The lncRNA colon cancer-associated transcript 1 (CCAT1) has been demonstrated to interact directly with upstream members of the AR pathway, potentially perpetuating the AR signal and promoting cell survival [433]. However, increased levels of CCAT1 and decreased levels of miR-24-3p have been observed in PTX-resistant CRPC tissues [434,435]. In vitro models have demonstrated that knockdown of CCAT1 or overexpression of miR-24-3p increase the sensitivity of resistant cells to PTX, while a miR-24-3p inhibitor disrupts the binding interaction and reverses the sensitivity [434]. Lastly, the upregulation of lncRNA urothelial carcinoma-associated 1 (UCA1) has been positively correlated with increased silent mating type information regulation 2 homologue 1 (Sirt1) and decreased miR-204 which, when combined, act in a regulatory manner to avoid drug-induced apoptosis [436,437]. siRNA inhibition of UCA1 and Sirt1 increased levels of miR-204, while the use of miR-204 mimics reduced Sirt1 levels [437]. The combination of the three further reduced DTX resistance in vitro, suggesting that all of the downstream pathways should be explored more to understand this additive response [437]. With the existence of multiple lncRNAs targeting different miRNAs, the concept of targeting one pathway to overcome resistance seems unlikely. However, more research should be done to understand ceRNAs with multiple targets or potential overlaps in target pathways.

There are also some lncRNAs that play a role in the response to taxanes without miRNA mediation. For example, the suppressor of cytokine signaling 2-antisense transcript 1 (SOCS2-AS1) is an androgen-related lncRNA that is overexpressed in long-term androgen-deprived CRPC cells compared to long-term androgen-dependent parental cells [438]. SOCS2-AS1 promotes AR signaling by suppressing apoptotic AR-target genes; knockdown of SOCS-AS1 upregulates TNF family genes and sensitizes cells to DTX, while overexpression of SOCS2-AS1 induces resistance [438]. HOXD-AS1 is also highly expressed in CRPC and the knockdown inhibited proliferation and reversed resistance to PTX in vitro and in vivo [439]. HOXD-AS1 correlates with response to taxanes by utilizing WD repeat domain 5 (WDR5) to act as the direct regulator of target genes such as PLK1, AURKA, CDC25C, UBE2C, and FOXM1, which all play roles in continuing the cell cycle [440,441,442]. Lastly, the lncRNA HORAS5 is associated with cell proliferation in CRPC and HORAS5-overexpressing cells decreased growth inhibition by CBZ treatment in a dose-dependent manner [443]. This suggests that CBZ stimulates the overexpression of HORAS5 or that the cells overexpressing HORAS5 are more primed to survive increasing concentrations of CBZ. Knockdown of HORAS5 confirmed the role in CBZ response, which demonstrated a CBZ concentration-dependent increase in growth inhibition [443]. Because there are so many lncRNAs that have different target pathways, using one specific inhibitor or ASO may not be the best approach to prevent or fight resistance, but monitoring levels of pro-survival lncRNAs could be used as a biomarker for patient response to taxanes.

### 4.6. Hypoxia Response Pathway

The relationship between hypoxia and taxane-resistant CRPC is well established but the precise protein interactions are not fully understood [303]. The transcription factor HIF1-α has been loosely associated with disease progression; HIF1-α knockdown decreases CRPC cell survival, whereas induction of HIF1-α improves cell survival [444]. The connection to taxane resistance, like in other cancers, mostly comes from the activation of other mechanisms. HIF1-α levels remaining constant could still allow for activation of downstream pathways to actively combat the current taxane treatment. In addition, upon PTX treatment in hypoxic conditions, HIF1-α co-localized with microtubules, immunoprecipitated with α-tubulin, and displayed binding to polymerized tubulin over soluble tubulin [445]. This could suggest that the PTX treatment inducing microtubule stabilization helped to traffic HIF1-α into the nucleus for protection from degradation and activation of pro-survival genes, thus priming the cell for reduced sensitivity to treatment. In addition, HIF1-α and activation of miR-210 correspond to continuation of hypoxia signaling and cellular alterations such as decreased mitochondrial metabolism and stem cell survival [446]. Low levels of miR-210 corresponded with lowered PSA levels following chemotherapy treatment, indicating an inverse relationship between response to treatment and miR-210/HIF1-α [447]. Hypoxic conditions also increased the EMT marker, vimentin, and decreased E-cadherin due to upregulated HIF1-α, but decreased vimentin and HIF1-α, protein levels following treatment with HIF1-α expression inhibitor, Propofol [448]. This suggests a potential method of action for successful HIF1-α therapeutic inhibitors, which in theory could help to restore sensitivity to taxanes. However, targeting HIF1-α may not be necessary due to variable basal HIF1-α levels depending on the tumor. In fact, DTX was found to remain effective at inducing cell death even in hypoxic conditions, implying that the basal levels of HIF1-α could act as a predictive biomarker of DTX sensitivity [449]. There are also alternatives to targeting HIF1-α while still inhibiting the hypoxic response. TH-302, a hypoxia-induced pro-drug (HAP), functions as a DNA alkylating agent to induce damage and cell death [450]. In vitro studies testing the combination of TH-302 with DTX produced a greater suppression of cell proliferation than either compound alone, and the combination demonstrated an increased delay in tumor growth in vivo compared to the agents individually [451]. However, if there is an upregulation of HIF1-α despite cellular oxygen availability, TH-302 and other HAPs might not be effective [452]. This information presents hypoxia and upregulated HIF1-α as possible contributors to taxane resistance in CRPC, yet more research must be done on the frequency of hypoxia and the basal HIF1-α levels of the tumor before considering them to be therapeutic targets.

## 5. Taxane Resistance in Other Cancers

In addition to breast, prostate, and ovarian cancers, taxanes are also used to treat a variety of other cancers. Drug resistance remains problematic, plaguing the potential for long-term success of taxane-based chemotherapeutics. Here, we review reported mechanisms of taxane resistance in non-small-cell lung, cervical, pancreatic, head and neck, and nasopharyngeal cancers.

### 5.1. Non-Small-Cell Lung Cancer

Non-small-cell lung cancer (NSCLC) accounts for ~85% of lung cancers and is typically treated with platinum-based drugs as a first-line defense [453]. Monotherapy, however, presents a high risk for tumor recurrence and patient relapse, making combination with a drug such as a taxane an attractive option. Despite this, taxane resistance can also occur in patients with NSCLC through many of the same mechanisms described above. Expression of lncRNAs can regulate drug efflux through MDR1 and the apoptotic response through the BCL-2 family of proteins (reviewed in [454]). NSCLC patients treated with both cisplatin and PTX had decreased miR-451 expression, increased metastasis, and poor prognosis [455]. miR-451 is negatively regulated by increased expression of Notch-1, and miR-451 targets the drug efflux pump MDR1 ([456] and reviewed in [453]). Taken together, these data suggest a promising role for miR-451, Notch signaling, and MDR1 in mediating taxane resistance in NSCLC.

As with other tumor types, the role of βIII-tubulin in taxane-resistant NSCLC has also been explored. Patients with low *TUBB3* expression, the gene encoding βIII-tubulin, have significantly increased response to treatment with platinum and PTX [457,458].

### 5.2. Cervical Cancer

As with NSCLC, platinums are the first-line monotherapy for cervical cancer; however, platinum and PTX combination therapy has proven superior [459]. miR-125a was significantly downregulated in PTX-resistant cervical cancer cells compared to the sensitive cells. Upregulation of miR-125a sensitized cells to PTX in vitro and PTX only in vivo through downregulating STAT3 and increasing drug-induced apoptosis [460]. Another study determined significant activation of the PI3K pathway in PTX-resistant cervical cancer. Therefore, PTX in combination with PI3K inhibition increased G2-M arrest in PTX-resistant cervical cancer cells, shown by inactivation of cyclin B1 and CDK1 [461].

There are seven G protein-coupled receptor kinases (GRKs), which constitute a class of protein kinases that can modify drug sensitivities through interactions with receptors and non-receptor proteins. GRK5 and HDAC6 form a signaling complex where GRK5 phosphorylates HDAC6 at Ser-21, promoting deacetylase activity, which may contribute to PTX resistance in cancer cells [97]. HeLa cervical cancer cells with reduced GRK5 protein expression show increased PTX sensitivity [98]. Results indicate that reduced GRK5 expression levels lead to decreased phosphorylation of HDAC6 at Ser-21, subsequently lowering HDAC6 activity. This suggests that treatments with PTX could be more successful among cancers with lower GRK5 protein expression. Additionally, the inclusion of a selective GRK5 inhibitor in combination with PTX could increase cancer cell death [462,463].

There is limited evidence supporting a possible relationship between the autophagy pathway and HIF1-α in taxane-resistant cervical cancer. PTX-resistant HeLa cells became sensitized to PTX when autophagy was inhibited (via ATG7 siRNA), HIF1-α siRNA or glycolysis inhibitors were used [464]. Because inhibiting glycolysis and knockdown of HIF1-α decreased autophagy [464], a relationship between the autophagy pathway and HIF1-α could be mediated by glycolysis or glycolysis-related proteins. More research should be done to investigate the role of HIF1-α in activating survival pathways following taxane treatment in cervical cancer.

### 5.3. Pancreatic Cancer

It is estimated that more than 57,000 cases of pancreatic cancer will be diagnosed in the US in 2020. While taxanes are typically used to treat advanced pancreatic cancer, combination therapies including a taxane have yielded promising results [465]. Gemcitabine, the standard of care for pancreatic cancer, used in conjugation with nab-PTX, has been studied widely and proven to be more effective than monotherapy ([465,466] and reviewed in [467]). Consistent with other cancers, βIII-tubulin plays a role in taxane-resistant pancreatic cancer. In vitro silencing of βIII-tubulin increased PTX sensitivity and reduced the tumorigenic potential of pancreatic cancer cells [36]. Additionally, there is evidence that NF-κB-mediated upregulation of anti-apoptotic BCL-2 family members is responsible for taxane resistance in some pancreatic cancers. Ectopic expression of NF-κB signaling molecules conferred resistance to Taxol while specific inhibition of NF-κB signaling sensitized pancreatic cancer cells to CBZ [468,469].

### 5.4. Head and Neck Cancer

Over half a million cases of head and neck squamous cell cancer (HNSCC) are reported annually and over 300,000 patients die from this disease worldwide every year [470,471]. While in its early stages, HNSCC is highly curable, the majority of patients are diagnosed with later stage cancer, which has a worse prognosis [470]. The current standard chemotherapy for HNSCC is cisplatin plus 5-fluorouracil (5-FU) [472], but clinical trials indicate that PTX and DTX can improve patient response and survival rates [470].

A study conducted by Zhang et al. reports that increased Notch1 expression in HNSCC is associated with PTX resistance. HNSCC with upregulated Notch1 showed increased resistance to PTX and DTX both in vitro and in vivo [470]. Use of the γ-secretase inhibitor, DAPT, to block the Notch signaling pathway improves sensitivity to anticancer drugs [470,473]. Overall, Notch1 expression is strongly associated with resistance to PTX and has potential as a biomarker for prognosis in HNSCC.

### 5.5. Nasopharyngeal Cancer

Nasopharyngeal cancer (NPC) is an invasive squamous cell cancer. A defining feature of NPC is its high sensitivity to radiotherapy, making it the current standard of treatment. However, over 60% of NPC patients are diagnosed with stage III or IV disease, for which prognosis following only radiotherapy is poor [474]. This has led researchers to investigate radiotherapy and chemotherapy drugs (such as PTX), as well as neoadjuvant therapy, to improve treatments for patients with later stage NPC. Although taxanes have revealed a possible advancement in treatment, chemoresistance remains an obstacle in improving patient prognosis, making research surrounding taxane resistance imperative for advancing patient treatment.

Similar to other cancer types, alterations in drug efflux proteins can result in taxane resistance. Knockdown of ABCC5 blocks drug efflux, allowing for increased intracellular PTX and induction of PTX-mediated cell death [475]. Furthermore, SNPs in the solute carrier, SLCO1B3, and the ATP-binding protein, ABCB1, lead to DTX response [476]. Interestingly, alterations in SLCO1B3 are also present in DTX resistance in prostate cancer, as described above [327,332].

Activation of signal transduction pathways and proteins involved in cell cycle has also shown to be important in taxane resistance in NPC, such as PLK1 [474], stathmin/ERK signaling [477], PI3K/Akt [478], MAPK signaling [479,480], and NF-κB/survivin [481].

Expression changes in non-coding RNAs also influence the response to taxanes in NPC. Expression of miR-1204, miR-634, or miR-29c, which silences ITGB1, sensitizes cells to taxanes [482,483,484]. The lncRNA CCAT acts as a sponge for miR-181a, and its knockdown sensitizes cells to PTX [485]. Another lncRNA, murine retrovirus integration site 1 homolog antisense RNA 1 (MRVI1-AS1), is decreased in PTX-resistant cell lines through the Hippo pathway [486]. Forced expression of MRVI1-AS1 sensitizes cells to PTX, and this method of taxane resistance also appears to be present in breast and lung cancers [486].

## 6. Concluding Remarks

Early taxanes and their later generations have been widely used since their clinical approval, are still effective first-line treatments for many epithelial-derived tumors, and are now being investigated in combination therapy (Table 4). However, many patients are either non-responsive initially or lose response over time due to resistance. Though taxane resistance is not fully understood, some mechanisms have become major players and are frequently observed even in different cancer types. Decreased intracellular drug concentration (due to increased drug export), increased metabolism of taxanes (a consequence of upregulating CYP enzymes), and altering tubulin subunit composition (specifically, the upregulation of βIII-tubulin) all culminate in reducing the effectiveness of taxanes’ method of action. When chemotherapy is at low levels or is unable to bind properly to the target, cells may be unable to arrest properly, and apoptosis is not induced. In addition, hypoxic conditions in solid tumors may play a larger role in activating these resistance pathways, depending on the cancer type. These common mechanisms are only some that exist in the pathway to resistance, and different pathways may also be altered in other cancer types. Monitoring individual protein or gene levels in tumors as biomarkers aids in correlating specific mechanisms to chemotherapy response or highlighting them as potential future targets of therapeutic inhibition (Table 5). Because every tumor is unique, a more individualized treatment plan with chemotherapy combined with an inhibitor to an altered pathway or an alternative chemotherapy may reverse resistance or could prevent it altogether.

## Figures and Tables

**Table 1:** Common resistance mechanisms. Mechanisms that are shared between more than 1 tumor type are grouped together here. See text for references.

Mechanism	Cancer Types	Taxane
**Taxane-Metabolizing Enzymes (Cytochrome p450 (CYP) family)**		
CYP3A4	Breast, Ovarian	PTX and DTX
CYP2C8	Ovarian	PTX
CYP1 enzymes (CYP1A1 and CYP1B1)	Ovarian	PTX
**Drug efflux/influx**		
MDR1	Breast, Ovarian, Prostate, Non-small cell lung cancer (NSCLC), Nasopharyngeal	PTX, DTX, and CBZ
ABCC10	Breast	PTX and DTX
ABCC5	Nasopharyngeal	PTX
Solute carrier of organic anions (SLCO)	Prostate, Nasopharyngeal	DTX
**Tubulin subunit and related protein expression**		
βIII-tubulin	Breast, Ovarian, Prostate, NSCLC, Pancreatic	PTX, DTX, and CBZ
Tau	Breast, Ovarian	PTX
Stathmin	Breast, Nasopharyngeal	PTX
**Signaling molecules**		
Polo-like kinase (PLK1)	Ovarian, Prostate, Nasopharyngeal	PTX
Bcl-XL/Bcl-2	Ovarian, Prostate, NSCLC, Pancreatic	PTX, DTX, and CBZ
Mcl-1	Ovarian, Prostate	PTX
PI3K/AKT pathway	Ovarian, Prostate, Cervical, Nasopharyngeal	PTX and DTX
G Protein-Coupled Receptor Kinase 5 (GRK5)	Breast, Cervical	PTX
Survivin	Ovarian, Nasopharyngeal	PTX

**Table 2:** Unique resistance mechanisms. See text for references.

Cancer Type	Mechanism	Taxane
**Breast**	MAP4	PTX
	Septin	PTX
	Tubulin Binding Cofactor C (TBCC)	PTX
	NIMA-related Kinase 2 (NEK2)	PTX
	G Protein Signaling Modulator 2 (GPSM2/LGN)	PTX
	BRCA1	PTX
	Adenomatous Polyposis Coli (APC)	PTX
	p16	PTX
	human Expanded (hEX)	PTX
	Yes-Associated Protein (YAP)	PTX
	Leucine Zipper Tumor Suppressor 1 (LZTS1)	PTX
**Ovarian**	Spleen Tyrosine Kinase (SYK)	PTX
	Cyclin E1 and CDK2	PTX
	Cyclin A1 and CDK1/CDK2	PTX
	BUB1	PTX
	BUBR1 (BUB1-related protein kinase)	PTX
	MAD2 (mitotic arrest deficiency 2)	PTX
	c-IAP1 and XIAP (X-linked IAP) overexpression	PTX
	Src	PTX
**Prostate**	Androgen Receptor (AR)	DTX and CBZ
**Pancreatic**	NF-kB	CBZ
**Head and Neck squamous cell carcinoma**	Notch signaling	PTX and DTX

**Table 3:** Non-coding RNAs involved in chemoresistance. The common miRNAs and lncRNAs are listed first. See text for references.

miRNAs	Cancer Type	Taxane
miR-451	NSCLC, Breast	PTX
miR-200 family (miR-141, miR-200c, and miR-200a)	Ovarian, Prostate, Breast	PTX, DTX
miR-17	Breast	PTX
miR-18a-5p	Breast	PTX
miR-18a	Breast	PTX
miR-20b	Breast	PTX
miR-21	Prostate	DTX
miR-29c	Nasopharyngeal	PTX
miR-34a	Prostate	PTX
miR-106a	Ovarian	PTX
miR-125b	Breast	PTX
miR-133b	Ovarian	PTX
miR-143	Prostate	DTX
miR-146	Ovarian	PTX
miR-148	Prostate	PTX
miR-194	Ovarian	PTX
miR-433	Ovarian	PTX
miR-520h	Breast	PTX
miR-591	Ovarian	PTX
miR-630	Ovarian	PTX
miR-634	Nasopharyngeal	PTX
miR-663	Breast	PTX
miR-1204	Nasopharyngeal	PTX
miR-1307	Ovarian	PTX
miR-3646	Breast	DTX
**lncRNAs**	**Cancer Type**	**Taxane**
Colon cancer-associated transcript 1 (CCAT1)	Prostate, Nasopharyngeal	PTX
Nuclear paraspeckle assembly transcript 1 (NEAT1)	Prostate, Ovarian	DTX
Urothelial carcinoma-associated 1 (UCA1)	Prostate, Ovarian	PTX
Ferritin like lnRNAs (FER1L4, FTH1P3)	Breast, Ovarian	PTX
AK124454	Breast	PTX
HIF1A-AS2	Breast	PTX
HORAS5	Prostate	CBZ
HOXD-AS1	Prostate	PTX
Long intergenic non-protein coding RNA 00518 (linc00518)	Prostate	PTX
LINC01118	Ovarian	PTX
lncRNA H19	Breast	PTX
Long intergenic non-coding RNA, Regulator of Reprogramming (Linc-ROR)	Breast	PTX
MT-associated protein tau antisense RNA 1 (MAPT)-AS1	Breast	PTX
MA-linc1	Breast	PTX
Murine retrovirus integration site 1 homolog antisense RNA 1 (MRVI1-AS1)	Nasopharyngeal	PTX
NONHSAT141924	Breast	PTX
Small nucleolar RNA host gene 6 (SNHG6)	Prostate	PTX
Suppressor of cytokine signaling 2-antisense transcript 1 (SOCS2-AS1)	Prostate	DTX

**Table 4:** Clinical trials of inhibitors to combat resistance.

Ref	Phase	Treatment	Prior Taxane	Cancer Type	Primary Outcomes
[265]	I	Dasatinib+ PTX OR carboplatin	N/A	Advanced and recurrent EOC	Recommended phase II dasatinib dose of 150mg daily with PTX and carboplatin
N/A	I	Pembrolizumab+ DTX OR gemcitabine hydrochloride	N/A	Urothelial cancer	Ongoing (NCT02437370)
[412]	I/II	Everolimus+ DTX	N/A	mCRPC	Recommended Everolimus dose of 10mg daily and DTX 60 mg/m^2^
[365]	I/II	CBZ+ abiraterone	DTX (I) and DTX+abiraterone (II)	mCRPC	Manageable safety profile and shows antitumor activity
[12]	II	Nab-PTX+ gemcitabine OR simplified LV5FU2	N/A	Metastatic pancreatic	N/A
[262]	II	Dasatinib	One or two regimens of platinum+taxane	EOC or primary peritoneal	Dastinib has minimal activity as a single agent in these cancers
[487]	II	DTX+ imatinib	N/A	metastatic BC	Weekly DTX is not enhanced by concurrent imatinib
[488]	II	LCL161+ PTX	N/A	TNBC	Toxicity concerns with combination
[351]	II	DTX OR CBZ+ prednisone	N/A	mCRPC	Improved prostate-specific antigen response rates
[399]	II	DTX+prednisone +placebo OR AT-101	N/A	mCRPC	Combination did not extend OS
[19]	III	CBZ+prednisone OR mitoxantrone+ prednisone	DTX-containing regimen	mCRPC	CBZ + prednisone improves OS
[465]	III	Nab-PTX+ gemcitabine	N/A	Metastatic pancreatic	Nab-PTX + gemcitabine improved OS

**Table 5:** Biomarkers of taxane resistance.

Biomarker	Cancer Types	Biomarker Status	Reference
Tau expression	Breast, Ovarian	Human Specimens	[80,181]
miRNAs and lncRNAs	Breast, Ovarian, Prostate		See text for individual references
βIII-tubulin	Ovarian, Prostate	Human Specimens	[193,338]
MDR1 and other ABC transporter proteins	Ovarian, Prostate	In vitro	[42,307,324,325] reviewed in [20]
Expression of CYP2C8, 3A4, 3A5	Ovarian	Human Specimens	reviewed in [20]
MAPs and MAPKs (IKBKB/STK39 and EDN2/TBK1)	Ovarian	In vitro	[185,186,187]
KIF14	Ovarian	In vitro, Human Specimens	[188]
CCNE1	Ovarian	In vitro	[201]
MAD2 (mitotic arrest deficiency 2)	Ovarian	In vitro, Human Specimens	[211,212,213]
PLK1 (Polo-like kinase 1)	Ovarian	In vitro	[201,219,220]
Inhibitor of apoptosis (IAP) family of proteins	Ovarian	Human Specimens, In vitro	[234,235,236,237,238]
Solute carrier of organic anions (SLCO)	Prostate	In vitro, Human Specimens	[326,327,328,331,332,334]
Increased Notch1 expression	HNSCC	Human Specimens	[470]
HIF1-α	Prostate	In vitro	[449]
